# fMRI connectivity as a biomarker of antipsychotic treatment response: A systematic review

**DOI:** 10.1016/j.nicl.2023.103515

**Published:** 2023-09-23

**Authors:** L.S. Dominicus, L. van Rijn, J. van der A, R. van der Spek, D. Podzimek, M. Begemann, L. de Haan, M. van der Pluijm, W.M. Otte, W. Cahn, C.H. Röder, H.G. Schnack, E. van Dellen

**Affiliations:** a Department of Psychiatry, Brain Center, University Medical Center Utrecht, Utrecht, The Netherlands; b Department Early Psychosis, Academical Medical Centre of the University of Amsterdam, Amsterdam, Amsterdam, The Netherlands; c Department of Radiology and Nuclear Medicine, Amsterdam UMC, University of Amsterdam, The Netherlands; d Department of Psychiatry, Amsterdam UMC, University of Amsterdam, The Netherlands; e Department of Child Neurology, UMC Utrecht Brain Center, University Medical Center Utrecht, and Utrecht University, Utrecht, The Netherlands; f Department of Intensive Care Medicine and UMC Utrecht Brain Center, University Medical Center Utrecht, Utrecht University, Utrecht, The Netherlands

**Keywords:** Psychosis, Prediction, Antipsychotic response, Functional connectivity, fMRI

## Abstract

**Background:**

Antipsychotic drugs are the first-choice therapy for psychotic episodes, but antipsychotic treatment response (AP-R) is unpredictable and only becomes clear after weeks of therapy. A biomarker for AP-R is currently unavailable. We reviewed the evidence for the hypothesis that functional magnetic resonance imaging functional connectivity (fMRI-FC) is a predictor of AP-R or could serve as a biomarker for AP-R in psychosis.

**Method:**

A systematic review of longitudinal fMRI studies examining the predictive performance and relationship between FC and AP-R was performed following PRISMA guidelines. Technical and clinical aspects were critically assessed for the retrieved studies. We addressed three questions: Q1) is baseline fMRI-FC related to subsequent AP-R; Q2) is AP-R related to a change in fMRI-FC; and Q3) can baseline fMRI-FC predict subsequent AP-R?

**Results:**

In total, 28 articles were included. Most studies were of good quality. fMRI-FC analysis pipelines included seed-based-, independent component- / canonical correlation analysis, network-based statistics, and graph-theoretical approaches. We found high heterogeneity in methodological approaches and results. For Q1 (N = 17) and Q2 (N = 18), the most consistent evidence was found for FC between the striatum and ventral attention network as a potential biomarker of AP-R. For Q3 (N = 9) accuracy’s varied form 50 till 93%, and prediction models were based on FC between various brain regions.

**Conclusion:**

The current fMRI-FC literature on AP-R is hampered by heterogeneity of methodological approaches. Methodological uniformity and further improvement of the reliability and validity of fMRI connectivity analysis is needed before fMRI-FC analysis can have a place in clinical applications of antipsychotic treatment.

## Highlights

• Antipsychotic treatment response emerges after weeks of therapy.
• Most evidence was found for FC between the striatum and ventral attention network.
• Heterogeneous methodological approaches hinder fMRI-FC literature.
• Improved reliability/validity is needed for fMRI-FC analysis in treatment.
• Refinement is required for clinical application of fMRI-FC.

## 1 Introduction

Antipsychotic drugs (AP) are the first-choice treatment for schizophrenia spectrum and other psychotic disorders, characterized by delusions, hallucinations, disorganized thinking, abnormal motor behavior (including catatonia), and negative symptoms (American Psychiatric Association, 2013). Worldwide, the lifetime prevalence of schizophrenia spectrum disorders is around 3% (Moreno-Küstner et al., 2018, Jonas et al., 2020). In up to 40% of patients, remission of symptoms is not achieved in response to the first prescribed AP (Meltzer, 1997). The success of treatment is currently unpredictable and only becomes clear after several weeks of therapy (Kahn et al., 2018). As a consequence, AP-treatment is based on a trial-and-error process (Leucht et al., 2013). A reliable marker for identifying individual antipsychotic treatment response (AP-R) could therefore significantly aid clinical care for patients with psychosis (Fond et al., 2015).

Functional connectivity (FC) in functional magnetic resonance imaging (fMRI) has been suggested as a potential AP-R biomarker. fMRI-FC can be characterized as the interdependency of fluctuations in low-frequency blood oxygen level-dependent (BOLD) signals from different brain regions (Biswal et al., 1995). fMRI-FC alterations have been associated with clinical impairment in patients with psychosis, and have been related to AP-R in cross-sectional studies (for systematic reviews of these studies, see (van den Heuvel and Fornito, 2014, Mwansisya et al., 2017). Treatment-resistant patients showed reproducible alterations in visual and auditory information processing regions and changes in sensorimotor network areas that were not present in patients responsive to AP (Chan et al., 2019). In contrast, AP-R was associated with activation of and deviated fMRI-FC within or between fronto-temporal, cortico-striatal, default mode and salience networks (Molent et al., 2019). These results support the concept of macroscale brain network alterations in patients with psychotic disorders, associated with AP-R. However, longitudinal designs are required to develop individualized biomarkers or outcome predictors, given that AP-R can only be measured after several weeks of treatment. A recent review of longitudinal fMRI studies in first-episode (FE) schizophrenia showed hypo-activation at baseline in various brain areas (i.e., prefrontal cortex, amygdala and hippocampus and basal ganglia), followed by normalization of brain activity after treatment (irrespective of AP-R) (González-Vivas et al., 2019). In more chronic schizophrenia samples, the most consistently reported finding is normalization or increased activation in frontal cortical regions (Kani et al., 2017). In addition, previous literature was also able to predict AP-R using machine learning techniques (Suvisaari et al., 2018, Mehta et al., 2021).

**Fig. 1:**
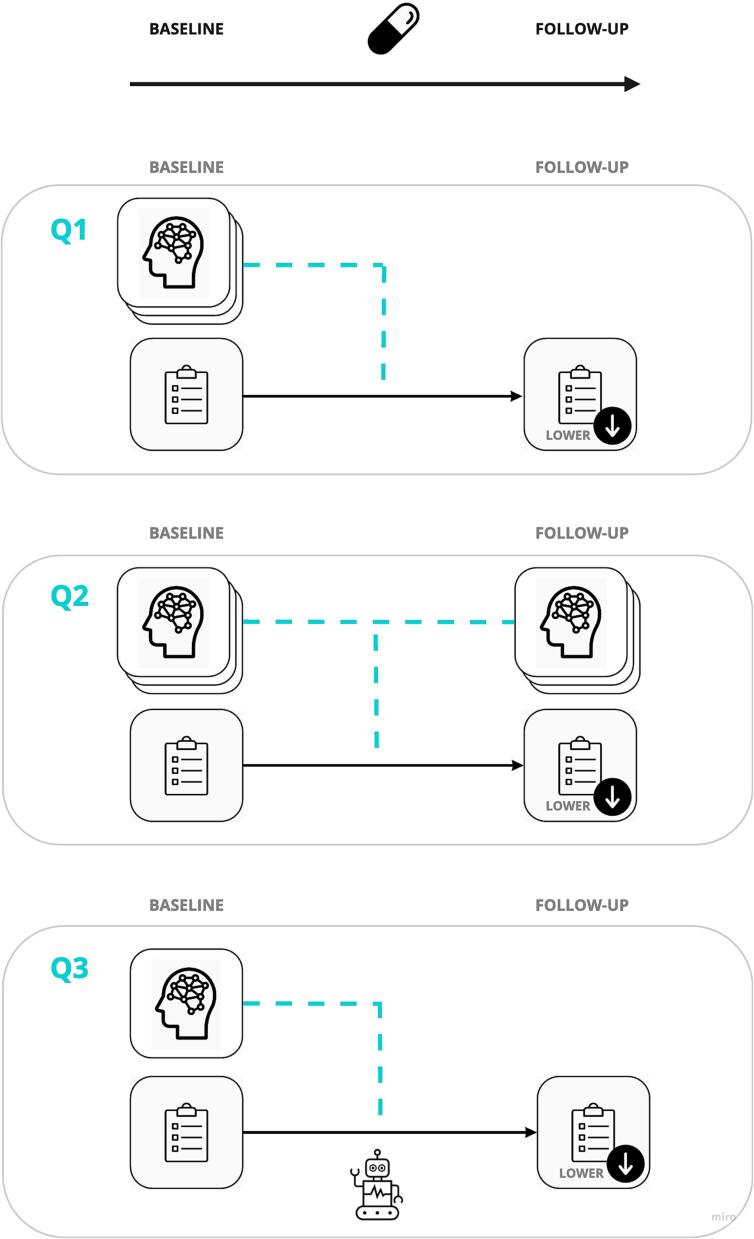
**Research questions** Overview research questions. Q1: Baseline FC related to AP-R, Q2: FC change over time related to AP-R, Q3: FC predicting AP-R, by using machine learning techniques.

In this systematic review, we aimed to evaluate the evidence that fMRI-FC is related to AP-R, and the potential of fMRI-FC to serve as biomarker of AP-R on an individual level. We therefore systematically reviewed the literature on 1) group level comparisons of fMRI-FC at baseline in relation to AP-R, 2) fMRI-FC change related to AP-R, and 3) fMRI-FC based prediction models of AP-R (Fig. 1) (Loth et al., 2021). From this perspective, the highest level of evidence that fMRI-FC is a meaningful substrate of AP-R would be alignment in results to all three research questions, i.e. that 1) fMRI-FC patterns differ in patients before treatment when comparing responders and non-responders on a group level; 2) these patterns ‘normalize’ in longitudinal treatment data related to treatment effect and 3) baseline patterns can be used as predictive markers of AP-R. In this way, we are investigating a consistent deviation FC at baseline, that is related to AP-R, normalizes after effective treatment and is also able to predict AP-R, where the ability to predict AP-R based on baseline patterns would be the ultimate goal when seeking a biomarker (Loth et al., 2021). Analyzing both moderators, which influence the intensity or direction of the relationship between the intervention and treatment outcome, and predictors, which impact treatment outcomes independently of the specific treatment, can provide a multi-dimensional view of fMRI-FC's role in AP-R (Kazdin, 2007).

Previous reviews have addressed only part of these research questions isolation (Kani et al., 2017, Suvisaari et al., 2018, Mehta et al., 2021, Tarcijonas and Sarpal, 2019), without a focus on AP-R (González-Vivas et al., 2019, Kani et al., 2017), without a systematic approach (Suvisaari et al., 2018, Tarcijonas and Sarpal, 2019), or without considering individual connections. The integration of all three research questions within the current review offers a unique and comprehensive perspective that is crucial when seeking a robust biomarker. Studies on AP-R based on fMRI-FC analysis have used different analysis paradigms (e.g., seed-based versus whole-brain independent component analysis [ICA]); these paradigms show only moderately strong correlations, even within the same dataset (Rosazza et al., 2012). We therefore also evaluated the generalizability of findings across methodological approaches. The complexity of fMRI-FC interactions with AP-R studied in this review warrants a reading instruction;

• A correlation with symptom improvement (Q1, Q2) will be referred to as a *correlation to AP-R*.
• At baseline (Q1), altered FC can either be negatively correlated (FC-) or positively correlated (FC +) with improvement of symptoms (AP-R).
• In longitudinal comparisons between FC and clinical symptom scores over time (Q2), the FC at baseline can either increase or decrease over time.
• Symptoms of schizophrenia spectrum and other psychotic disorders can be categorized into different domains: positive symptoms (e.g., hallucinations and delusions) and negative symptoms (e.g., apathy). These will be referred to as PANSS/BPRS+ or PANSS/BPRS- respectively. Most AP primarily target the dopaminergic system, and their primary effect is the reduction of positive symptoms, with notable exceptions such as clozapine (Stahl, 2020, Golan et al., 2012). Since we wanted to provide a complete review of the literature, this review covers all symptoms (positive(+), negative(-), general, total scores).

## 2 Methods

### 2.1 Search strategy and selection criteria

A literature search was conducted according to Preferred Reporting Items for Systematic Reviews (PRISMA) guidelines (Flow Diagram, 2009). Studies published until October 2022 were included, using PubMed and EMBASE databases (for exact search terms, see supplementary file S1). Reference lists from the selected studies were reviewed for additional articles of relevance. The included studies met the following criteria: (a) examined participants with psychosis spectrum diagnoses by standard diagnostic criteria; (b) implement treatment with AP for; (c) any disease duration and current treatment status (medication-naive or currently using AP at baseline); (d) outcomes of fMRI-FC or functional network characteristics during a task or during resting-state (rs); (e) at least one scan performed at baseline; (f) at least two clinical assessments performed at different times with any interval between them; (g) comparisons included contrasts between AP-R and AP-NR (differentiation in AP-R); (h) articles published in English language. A cross-sectional study design was an exclusion criterion. To reduce the risk of selection bias, the literature search and the selection process were performed independently by two researchers (LvR, LD). A third author (EvD) was involved if no consensus was reached. Results were categorized by research question, i.e., Q1) baseline fMRI-FC related to AP-R; Q2) AP-R related change in fMRI-FC; and Q3) fMRI-FC as predictor of AP-R (Fig. 1). In instances where a study tackled multiple research questions, it was encompassed within several distinct research inquiries.

### 2.2 Data extraction

Information was recorded for each study on: (a) first author and year of publication; (b) study design; (c) fMRI features, including analytical approach (seed-based vs whole brain and paradigm (rs vs task-based); (d) sample characteristics, including number of subjects, diagnosis, age and gender; (e) type, dose (which, for comparability between studies, was calculated into a Daily Defined Dose (DDD) using olanzapine as reference) (Anon and Index, 2020, Leucht et al., 2016) and duration of AP-treatment; (f) AP-R outcome; (g) brain regions evaluated; (h) corresponding resting state network (RSN) (i.e. default mode network (DMN), dorsal attention network (DAN), frontoparietal network (FN), limbic network (LN), somatosensory network (SN), striatum, thalamus, ventral attention network (VAN), visual network (VN)) (For the seed-based studies that did not use a RSN framework, seeds were categorized into a RSN based on the estimated location defined by a previous whole-brain network parcellation in 1000 participants (Thomas Yeo et al., 2011); (i) fMRI-FC correlation with improvement of psychotic symptom score as defined in the introduction; (j) for fMRI-FC longitudinal studies: direction of FC change between seed-regions (i.e. increase or decrease of FC strength). Data were assessed separately for studies on 1) baseline fMRI-FC correlations with AP-R; 2) AP-R related change in fMRI-FC; and 3) fMRI-FC as predictor of AP-R. Detailed methods and results are provided in supplementary materials. These include extra information about the search strategy, calculation of DDD, Risk of Bias (RoB) -tool.

### 2.3 Data processing and Visualization

Initially, all significant neural connections identified in the dataset were tabulated. Next, a connectivity matrix was generated in Rstudio (Martin, 2021). To transform this structured data into a visual representation, the connectivity matrix was loaded into the Neuro Mapping and Visualization tool (NeuroMArVL) (Dwyer et al., 2017). By putting our data into this platform, we were able to generate detailed and informative visualizations of the significant neural connections, offering both a holistic overview and nuanced insights into the intricate interrelations present in the included studies.

### 2.4 Quality assessment

For all included studies, we critically assessed validity and generalizability aspects of the methodology of fMRI-FC analysis. We developed a scoring system summarizing relevant study characteristics that influenced the methodological quality of a study based on fMRI literature (Supplementary file S4). We evaluated both clinical study characteristics and methodological fMRI processing steps. These criteria were based on previous literature and tailored to the articles as included in our review (Lueken et al., 2016, van Montfort et al., 2019, Patriat et al., 2013, Ciric et al., 2017). Regarding the clinical parameters, our work was based on previous tools for finding a biomarker (Lueken et al., 2016) and the methodological aspects were based on state of art methodological studies (van Montfort et al., 2019). Clinical parameters and methodological fMRI parameters categories were quantified and a summative score was calculated. Clinical parameters included sample information, confounder control and outcome measures. Methodological fMRI parameters included motion correction, duration of scanning and information regarding the eyes during scanning (open, closed, not given). Two authors (LvR and LD) evaluated each study independently based on these. A third author (EvD) evaluated the study if the scores differed between authors, and the quality score was determined after consensus.

## 3 Results

### 3.1 Search results

The initial search yielded 4539 hits and 3749 articles without duplicates (Supplementary file S3). After screening and eligibility assessment 28 studies were included. In one study (Nelson et al., 2022) we identified two distinct cohorts, which we subsequently referred to as Nelson 2022a and Nelson2022b in our study. A total of 952 patients were investigated of which 551 with FE psychosis, 220 with two or more episodes or chronic psychosis, and 181 patients with unknown state of disease. Study characteristics are shown in Table 1a, Table 1b, Table 1c. Q1) seventeen studies reported on baseline fMRI-FC and AP-R at follow-up (Anticevic et al., 2015, Blazer et al., 2022, Cadena et al., 2019, Deng et al., 2022, Doucet et al., 2020, Hadley et al., 2014, Han et al., 2020, Kraguljac et al., 2016, Kraguljac et al., 2016, Li et al., 2020, Maximo et al., 2021, Nelson et al., 2022, Sarpal et al., 2016, Wang et al., 2019, Zhang et al., 2019, Blessing et al., 2020, Zong et al., 2019), Q2) eighteen studies investigated associations between changes in fMRI-FC characteristics before and after treatment related to AP-R (Anticevic et al., 2015, Blazer et al., 2022, Cadena et al., 2019, Chopra et al., 2021, Deng et al., 2022, Duan et al., 2020, Hadley et al., 2014, Hadley et al., 2016, Han et al., 2020, Kraguljac et al., 2016, Li et al., 2016, Li et al., 2020, Liu et al., 2022, Sarpal et al., 2015, Shan et al., 2021, Wang et al., 2019, Blessing et al., 2020, Zong et al., 2019), Q3) nine studies investigated the prediction of AP-R based on fMRI-FC (Cao et al., 2018, Li et al., 2020, Li et al., 2020, Liu et al., 2022, Sarpal et al., 2016, Shan et al., 2021, Zhu et al., 2018, Blessing et al., 2020, Nejad et al., 2013).

**Table 1a:** Overview of study characteristics baseline (Q1).

***First author, year of publication***	***Design and scan interval***	**Analytical approach**	***Participants (N)***	***Brain region of interest***	***Population***				***Treatment***		***Clinical assessment***
					***Disease state***	***Diagnosis***	***Mean age (SD) (years)***	***Gender (M/F)***	Antipsychotic (type) *	Mean dose (SD) mg/day****	
Anticevic, 2015	2 scans 12 months	seed-based	25	prefrontal cortex	FE chronic	Schizophrenia	23.60(9.22)	5/20	NR	5.6(6.2)	PANSS
Blessing, 2019	2 scans 8 weeks	ICA	27	hippocampus, whole-brain cortex	FE	Schizophrenia, schizophreniform disorder	24.11(7.19)	12/15	mixed (single medication N = 21, two medications N = 6)	16.3(9.2)	BPRS SANS
Blazer, 2022	2 scans 12 weeks	seed-based	22	striatum, DMN, FN, salience network	chronic	Schizophrenia, schizoaffective disorder	35 (9.2)	16/6	clozapine	18.2(15.8)	BPRS
Cadena, 2018	2 scans 6 weeks	seed-based***	22	FN (Connections with ACC)	chronic	Schizophrenia(N = 19), schizoaffective disorder(N = 3)	33(9.78)	17/5	risperidone	8.3(3.8)	BPRS RBANS
Deng, 2022	2 scans 6–24 months	seed based	47	DMN	FE	Schizophrenia		NA	mixed	9.7 (6.7)*****	SAPS SANS
Doucet, 2018	1 scan 24 weeks	CCA	76	DMN, VAN, CEN, SN, VN	chronic	Schizophrenia	26.9 (7.0)	19/57	mixed	9.3 (10.6)	BPRS
Hadley, 2013	2 scans 1 week**	seed-based	21	VTA/midbrain	FE (N = 6), chronic (N = 15)	Schizophrenia (N = 18), Schizoaffective disorder(N = 3)	36.0(10.2)	17/4	risperidone	NR	BPRS RBANS
Han, 2020	2 scans 8 weeks	seed-based	37	DAN, striatum	FE	Schizophrenia	25.05(5.01)	24/13	risperidone	8–12	PANSS
Kraguljac, 2016	2 scans 6 weeks	seed-based	34	hippocampus	FE(N = 12), chronic	Schizophrenia(N = 31), Schizoaffective disorder(N = 3)	32.38(10.43)	23/11	risperidone	8.7(2.9)	BPRS
Kraguljac, 2015	2 scans 6 weeks	seed-based	34	DAN, CEN, VAN, DMN	FE(N = 12), chronic	Schizophrenia(N = 31), Schizoaffective disorder(N = 3)	32.38(10.43)	23/11	risperidone	8.7(2.9)	BPRS
Nelson, 2022a	2 scans 6 weeks	seed- based	71	striatum	FE	NR	23.73 (6.00)	45/26	risperidone	8.8(4.4)	BPRS
Nelson, 2022b	2 scans 6 weeks	seed- based	42	striatum	Chronic	Schizophrenia	27.64(9.61)	30/12	risperidone	7.8(3.3)	BPRS
Maximo, 2021	1 scan 16 weeks	seed- based	52	DMN	FE	Schizophrenia (N = 26) Schizoaffective disorder(N = 12) Bipolar disorder (N = 3) Schizophreniform disorder (N = 2) Psychosis NOS (N = 7) Brief psychotic disorder (N = 1) Major depressive disorder with psychosis (N = 1)	24.46 (6.34)	33/19	risperidone	8.44(5.4)	BPRS
Sarpal, 2016	2 scans 12 weeks	seed-based	41	striatum	FE	Schizophrenia, schizophreniform disorder, schizoaffective disorder, psychotic disorder NOS	AP-R 21.2 (3.8) AP-NR: 21.9 (5.9)	29/12	risperidone(N = 22), aripiprazole(N = 18)	AP-R: 13.3(4.6) AP-NR:13.(6.7)	BPRS CGI
Wang, 2019	2 scans 4 months	seed-based	36	whole brain	FE	Schizophrenia	16–59(24.4 ± 8.8)	20/16	mixed (partial patients received multidrug therapy)	0–26.3 (7.8 ± 5.3)	PANSS
Zhang, 2019	2 scans 8 weeks	ICA	33	SN subnetworks	chronic	Schizophrenia	24.00(5.49)	10/13	NR	NR	BPRS SANS
Zong, 2018	2 scans 8 weeks	ICA	42	DMN	FE	Schizophrenia	24.86 (4,80)	27/15	risperidone	8.0–12	PANSS

* Mixed: ≥ 2 types of antipsychotics prescribed.

**Extra clinical assessment in week 6.

*** Task-**based**: Stroop-based color naming task.

**** Olanzapine equivalent daily dose.

***** Mean dose **calculated** based on highest dose of chlorpromazine per participant.

**Abbreviations:** ICA: independent component analysis, CCA: canonical correlation analysis, AP-R: antipsychotic response, AP-NR: antipsychotic non-response, NA: not applicable, FN: frontal-parietal network, ACC: anterior cingulate cortex, DMN: default mode network, VAN: ventral attention network, CEN: central executive network SN: sensorimotor network, VN: visual network, VTA: ventral tegmental area, DAN: dorsal attention network, FE: first-episode, NOS: not otherwise specified, DSM: Diagnostic and Statistical Manual of Mental Disorders, DIGS: Diagnostic Interview of Genetic Studies, SD: Standard deviation, M: male, F: female, NR: not reported, mg: milligram, BPRS: Brief Psychiatric Rating Scale, BPRS+: Brief Psychiatric Rating Scale, positive symptoms, BPRS-: Brief Psychiatric Rating Scale, negative symptoms, PANSS+: Positive and Negative Syndrome Scale, positive symptoms, PANSS -: Positive and Negative Syndrome Scale, negative symptoms, SANS: Scale for the Assessment of Negative Symptoms, RBANS: Repeatable Brief Assessment of Neuropsychological Status, CGI: Clinical Global Impression.

**Table 1b:** Overview study characteristics change (Q2).

***First author, year of publication***	***Design, scan interval***	***Analytical approach***	***Total participants (N)***	***Brain region of interest***	***Population***				***Treatment***		***Clinical assessment***
					***Disease state***	***Diagnosis***	***Mean age (SD) (years)***	***Gender (M/F)***	***Antipsychotic (type)***	Mean dose (SD) mg/day*****	
Anticevic, 2015	2 scans 12 months	seed-based	25	prefrontal cortex	FE Chronic	Schizophrenia	23.60(9.22)	5/20	NR	5.6 (6.2)	PANSS
Blazer, 2022	2 scans 12 weeks	seed-based CCA	22	striatum, DMN, FN, Salience	Chronic	Schizophrenia	35 (9.2)	16/6	clozapine	9.6 (12.4)	BPRS
Blessing, 2019	2 scans 8 weeks	ICA	27	hippocampus, cortex	FE	Schizophrenia, schizophreniform disorder	24.11(7.19)	12/15	mixed (single medication N = 21, two medications N = 6)	16.3(9.2)	BPRS SANS
Cadena, 2018	2 scans 6 weeks	seed-based**	22	FN (Connections with ACC)	Chronic	Schizophrenia(N = 19), schizoaffective disorder(N = 3)	33(9.78)	17/5	risperidone	8.3(3.8)	BPRS RBANS
Chopra, 2021	3 scans 3–12 months	NBS	28	whole brain	FE	Psychotic spectrum disorder (Major depression with psychosis N = 5) Schizophreniform disorder (N = 4), Psychotic disorder NOS (N = 7), Substance induced psychotic disorder (N = 2), Delusional disorder (N = 4), Schizophrenia (N = 5), Missing diagnosis (N = 1)	19.5 (3.0)	15/13	NR + intensive psychosocial treatment	NA	BPRS SOFAS
Deng, 2022	2 scans 6–24 months	seed-based	47	DMN	FE	Schizophrenia	NA	NA	mixed	9.7(6.7)****	SAPS SANS
Duan, 2020	2 scans 8 weeks	seed-based	33	DMN	FE	Schizophrenia	25.03 (4.71)	21./12	risperidone	Initially: 2–4 Max: 12	PANSS
Hadley, 2013	2 scans 1 week***	seed-based	21	VTA/Midbrain	FE (N = 6), Chronic (N = 18)	Schizophrenia	36.0 (10.2)	17/4	risperidone	NR	BPRS RBANS
Hadley, 2016	2 scans 6 weeks	graph theory	32	whole brain	FE (N = 10), Chronic (N = 22)	Schizophrenia(N = 29), Schizoaffective disorder(N = 3)	33.60 (10.38)	23/9	risperidone	AP-R: 8.1(2,6) AP-NR: 10.3 (2.1)	BPRS
Han, 2020	2 scans 8 weeks	seed-based	37	VAN, wtriatum	FE	Schizophrenia	25.05 (5.01)	24/13	risperidone	8.0–12	PANSS
Kraguljac, 2016	2 scans 6 weeks	seed-based	34	hippocampus	FE(N = 12)Chronic	Schizophrenia(N = 31), Schizoaffective disorder(N = 3)	32.38 (10.43)	67,6% male	risperidone	8.7(2.9)	BPRS
Li, 2020	2 scans 1 week****	seed-based	32	ACC	FE	Schizophrenia	30.94 (8.25)	16/16	olanzapine	10–30	PANSS
Li, 2016	2 scans 12 months	seed-based	20	whole brain	FE	Schizophrenia	22.9 (8.5)	6/14	mixed	8.0	PANSS GAF
Liu, 2022	2 scans 16 weeks	graph-theory	38	whole brain	FE Chronic	Schizophrenia	24.1 (8.6)	21/17	mixed	15.71(4.91)	PANSS
Sarpal, 2015	2 scans 12 weeks	seed-based	41	striatum	FE	Schizophrenia, schizophreniform disorder, schizoaffective disorder, psychotic disorder NOS)	AP-R 21.2(3.8) AP-NR: 21.9(5.9)	6/35	risperidone(N = 22), aripiprazole(N = 18)	AP-R: 13.3(4.6) AP-NR: 13.2(6.7)	BPRS CGI
Shan, 2021	2 scans 8 weeks	seed-based	20	whole brain	FE Chronic	Schizophrenia	22.75 (4.28)	15/5	olanzapine	20.50 (1.54)	PANSS
Wang, 2019	2 scans 4 months	graph theory, seed- based	36	whole brain	FE	Schizophrenia	16–59 (24.4 ± 8.8)	20/16	mixed	0–26.3 (7.8 ± 5.3)	PANSS
Zong, 2018	2 scans 8 weeks	ICA	42	DMN	FE	Schizophrenia	24.86 (4.80)	27/15	risperidone	8.0–12	PANSS

* **Mixed**: ≥ 2 types of antipsychotics prescribed.

** Task-based: stroop-based color naming task.

*** Extra clinical assessment in week 6.

**** Extra clinical assessment in week 8.

***** olanzapine equivalent daily dose.

**Abbreviations:** ICA: independent component analysis, NBS: Network-based statistics, CCA: canonical correlation analysis, AP-R: antipsychotic response, AP-NR: antipsychotic non-response, NA: Not applicable, FN: frontalparietal network, ACC: anterior cingulate cortex, DMN: default mode network, VAN: ventral attention network, CEN: central executive network SN: sensorimotor network, VN: visual network, VTA: ventral tegmental area, DAN: dorsal attention network, FE: first-episode, NOS: Not Otherwise Specified, DSM: Diagnostic and Statistical Manual of Mental Disorders, DIGS: Diagnostic Interview of Genetic Studies, SD: Standard deviation, M: male, F: female, NR: Not reported, mg: milligram, BPRS: Brief Psychiatric Rating Scale, BPRS+: Brief Psychiatric Rating Scale, positive symptoms, BPRS-: Brief Psychiatric Rating Scale, negative symptoms, PANSS+: Positive and Negative Syndrome Scale, positive symptoms, PANSS -: Positive and Negative Syndrome Scale, negative symptoms RBANS: Repeatable Brief Assessment of Neuropsychological Status, SAPS: Scale of the Assessment of Positive Symptoms, SANS: Scale of the Assessment of Negative Symptoms, CGI: Clinical Global Impression, DDD: Defined daily dose.

**Table 1c:** Overview study characteristics prediction (Q3).

***First author, year of publication***	***Design and scan interval***	***Analytical approach***	***Total participants (N)***	***Brain region of interest***	***Population***				***Treatment***		***Clinical assessment***
					***Disease state***	***Diagnosis***	***Mean age (SD) (years)***	***Gender (M/F)***	***Antipsychotic (type)***	Mean dose (SD) mg/day****	
Blessing, 2019	2 scans 8 weeks	ICA	27	hippocampus, cortex	FE	Schizophrenia, schizophreniform disorder	24.11(7.19)	12/15	mixeds ingle medication N = 21, two medications N = 6)	8.5	BPRS SANS
Cao, 2018	2 scans 10 weeks	seed-based	43	superior temporal cortex	FE	Schizophrenia	28.3 (9.9)	19/24	risperidone	6.0 – 12	PANSS
Li (Ang), 2020	1 scan PKU6: 6.5 (0,69), ZMD: 6,41 (0,57)	seed-based	95	striatum	NR	Schizophrenia	29.30 (7,86)	95/39	mixed	18****	PANSS
Li, 2020	2 scans 1 week**	seed-based	32	ACC	FE	Schizophrenia	30.94 (8.25)	16/16	olanzapine	10–30	PANSS
Liu, 2022	2 scans 16 weeks	graph -theory	38	whole brain	FE Chronic	Schizophrenia	24.1(8.6)	21/17	mixed	15.71(4.91)	PANSS
Nejad, 2012	1 scan 29.96 (2.48) weeks	ICA***	14	working memory networks	FE	Schizophrenia	AP-R: 23.93(3.76) AP-NR: 28.92(4.89)	11/3	quetiapine	12.96 (7.275)	PANSS
Sarpal, 2016	2 scans 12 weeks	seed-based	41	striatum	FE	Schizophrenia, schizophreniform disorder, schizoaffective disorder, psychotic disorder NOS	AP-R: 21.2 (3.8) AP-NR: 21.9 (5.9)	29 /12	risperidone(N = 22), aripiprazole(N = 18)	AP-R: 13.3(4.6) AP-NR: 13.2(6.7)	BPRS CGI
Shan 2021	2 scans 8 weeks	voxel mirrored homotopic connectivity	20	superior/middlemedial prefrontal cortex	FE Chronic	schizophrenia	22.75(4.28)	15/5	olanzapine	20.50(1.54)	PANSS
Zhu, 2018	2 scans 8 weeks	ICA	44	whole brain	FE	Schizophrenia	23.45 (4.24)	28/16	olanzapine	18.3 (5.2)	PANSS

*Mixed: ≥ 2 types of antipsychotics prescribed.

** Extra clinical assessment week 8.

*** Task-based: N-back task.

**** Olanzapine equivalent daily dose.

**** Mean dose calculated based on highest dose of chlorpromazine per participant.

**Abbreviations:** ICA: independent component analysis, CCA: canonical correlation analysis, AP-R: antipsychotic response, AP-NR: antipsychotic non-response, NA: Not applicable, FN: Frontalparietal network, ACC: Anterior cingulate cortex, DMN: default mode network, VAN: Ventral attention network, CEN: Central executive network SN: Sensorimotor network, VN: Visual network, VTA: Ventral tegmental area, DAN: Dorsal attention network, FE: First-episode, NOS: Not Otherwise Specified, DSM: Diagnostic and Statistical Manual of Mental Disorders, DIGS: Diagnostic Interview of Genetic Studies, SD: Standard deviation, M: male, F: female, NR: Not reported, mg: milligram, BPRS: Brief Psychiatric Rating Scale, BPRS+: Brief Psychiatric Rating Scale, positive symptoms, BPRS-: Brief Psychiatric Rating Scale, negative symptoms, PANSS+: Positive and Negative Syndrome Scale, positive symptoms, PANSS -: Positive and Negative Syndrome Scale, negative symptoms RBANS: Repeatable Brief Assessment of Neuropsychological Status, CGI: Clinical Global Impression.

### 3.2 ROB assessment and methodological quality

**Fig. 2:**
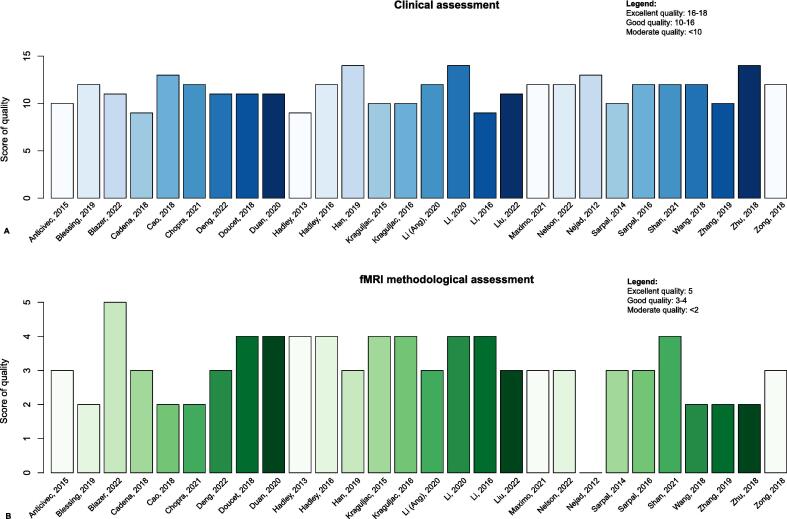
Overview ROB assessment score per study A: Clinical parameters, B: fMRI methodological parameters. On the X-axis the first author and year of the different studies. On the y-axis the total points given to the studies are shown. We considered the study Nelson et al. (Nelson et al., 2022) (nelson a and b) as one study for the ROB tool.

The quality of evidence was generally good for both clinical and methodological assessment (Fig. 2, suppl. Table S4). 11% of the studies scored moderate quality on the clinical assessment. Medication dosage and concomitant medication use were most often scored as suboptimal. 25% of studies were labeled as moderate quality on fMRI processing, most frequently due to suboptimal scan duration and the lack of reporting the rs condition. There was notable heterogeneity of populations between studies. Some of the studies focused exclusively on AP-naive patients (Anticevic et al., 2015, Cao et al., 2018, Chopra et al., 2021, Deng et al., 2022, Duan et al., 2020, Han et al., 2020, Li et al., 2016, Zhu et al., 2018, Blessing et al., 2020, Zong et al., 2019), whereas the others, included participants that where already medicated with AP before.

### 3.3 Q1: FC at baseline related to AP-R

Seventeen studies reported a difference in baseline FC between AP-R and AP-NR (Nelson et al., 2022, Anticevic et al., 2015, Blessing et al., 2020, Cadena et al., 2019, Doucet et al., 2020, Hadley et al., 2014, Han et al., 2020, Kraguljac et al., 2016, Kraguljac et al., 2016, Sarpal et al., 2016, Li et al., 2020, Wang et al., 2019, Zhang et al., 2019, Zong et al., 2019, Deng et al., 2022, Blazer et al., 2022, Maximo et al., 2021). Studies included 585 patients of which 408 FE patients and 177 chronic patients. Sixteen studies used a rs design, and one study used a task-based design (during a stroop-color-naming task) (Cadena et al., 2019). The interval between baseline and follow-up varied from 1 week to 24 months (median of eight weeks). Fifteen studies defined AP-R as a percentage of change in clinical symptom score or subscale score (positive or negative score). Three studies defined AP-R as a priori set percentage (i.e., 20, 30 and, 35%) reduction in symptom score (Blessing et al., 2020, Deng et al., 2022, Sarpal et al., 2016). Treatment consisted of risperidone monotherapy (N = 9 studies (Nelson et al., 2022, Cadena et al., 2019, Hadley et al., 2014, Han et al., 2020, Kraguljac et al., 2016, Kraguljac et al., 2016, Zong et al., 2019, Maximo et al., 2021) monotherapy with variable AP-agents (N = 4 (Blessing et al., 2020, Doucet et al., 2020, Wang et al., 2019, Blazer et al., 2022), monotherapy or multitherapy with variable AP-agents (Deng et al., 2022) or 1:1 randomization between risperidone and aripiprazole (Sarpal et al., 2016). Two studies did not specify the type of AP-treatment (Anticevic et al., 2015, Zhang et al., 2019). Regarding fMRI analysis, twelve studies used a seed-based approach, three studies used ICA (Blessing et al., 2020, Zhang et al., 2019, Zong et al., 2019), two studies used canonical-correlation analysis (CCA) (Doucet et al., 2020, Blazer et al., 2022), and one study used graph theoretical analysis (Wang et al., 2019).

**Fig. 3:**
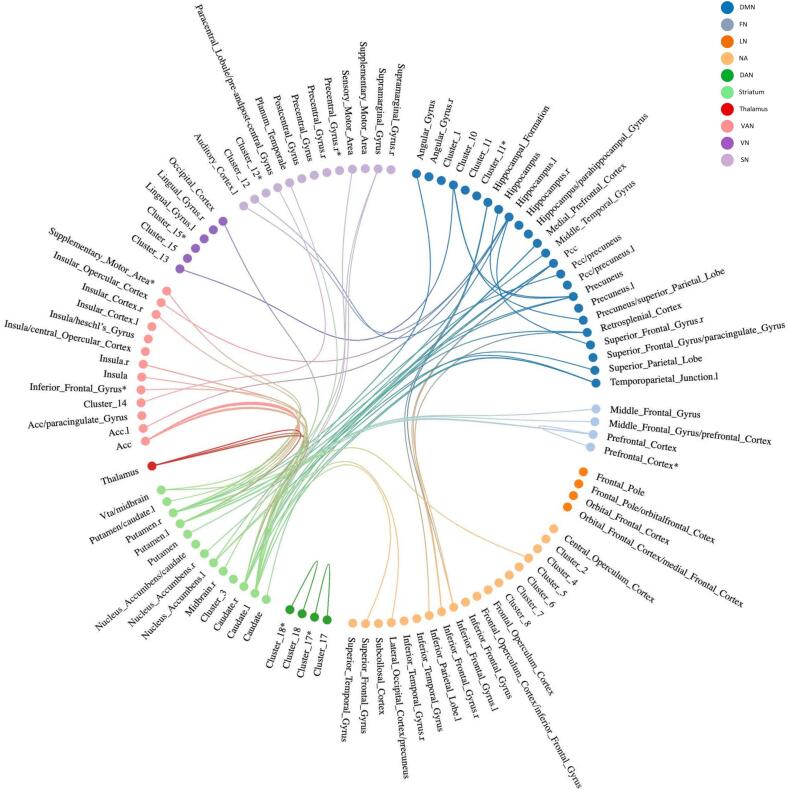
Overview of significant baseline FC + connections related to AP-R (Q1), indicating that higher baseline FC for these connections was associated with AP-R. The regions of each significant connection were divided in RSN, shown in corresponding color. If connections existed of more than two brain localizations, they were summarized into clusters If it was not possible to categorize regions of a significant connection in the RSN framework, it was assigned to the NA group. *Connection with itself. ***For an overview of the clusters see*** supplementary table S7***.* Abbreviations:** DMN: default mode network, FN: frontoparietal network, LN: limbic network, NA: not applicable, SN: sensorimotor network, VAN: ventral attention network, VN: visual network, ACC: anterior cingulate cortex, l: left, PCC: posterior cingulate cortex, r: right, VTA: ventral tegmental area.

**Fig. 4:**
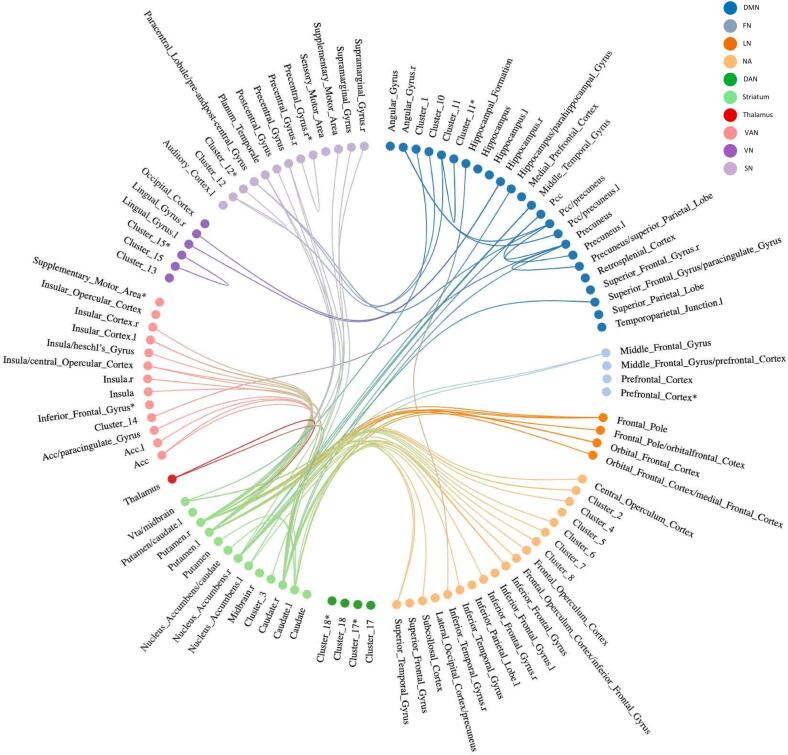
Overview of significant baseline FC- connections related to AP-R, indicating that lower baseline FC for these connections was associated with AP-R. Each significant connection is divided in RSN, shown in corresponding color. If connections existed of more than two brain localizations, they were summarized into clusters. If it was not possible to categorize regions of a significant connection in the RSN framework, it was assigned to the NA group. ***For an overview of the clusters see*** supplementary table S7***.*** *Connection with itself **Abbreviations:** DMN: default mode network, FN: frontoparietal network, LN: limbic network, NA: Not applicable, SN: sensorimotor network, VAN: ventral attention network, VN: visual network, ACC: anterior cingulate cortex, l: left, PCC: posterior cingulate cortex, r: right, VTA: ventral tegmental area.

At baseline a total of 117 unique connections related to AP-R (Fig. 3 and Fig. 4, supplementary file S5) were reported**.** The graph theoretical study showed no significant difference between AP-R and AP-NR. Six unique connections associated with AP-R were reported in more than one study (Table 2a). Most conclusive evidence was found in FC between striatum - VAN; these findings are reported in 235 patients, after treatment with risperidone (N = 194), clozapine (N = 22) or aripiprazole (N = 18) with clinical evaluation between 6 and 12 weeks follow-up. Three studies reported that putamen – anterior cingulate cortex (ACC) FC is related to AP-R; while in rs studies FC-dysconnectivity in this connection was negatively associated with AP-R (for positive symptoms) (Han et al., 2020, Sarpal et al., 2016), the task-based study showed an opposite result of FC-dysconnectivity positively associated with AP-R (for positive symptoms) (Cadena et al., 2019). Two studies reported that caudate - ACC is related to AP-R (Nelson et al., 2022, Sarpal et al., 2016), in this connection both a negative and positive association with AP-R was found (for positive symptoms).

**Table 2a:** Overview unique baseline connections related to AP-R described by two or more studies, categorized by RSN and connections (Q1).

**First author, year of publication**	**Analytical approach**	**Direction of correlation with symptom improvement**	**Clinical assessment**	**Region A, corresponding RSN**	**Region B, corresponding RSN**
				**striatum**	**VAN**
Han 2019, Sarpal 2016	seed-based	FC-	BPRS+, PANSS+	putamen	ACC
Cadena, 2018	seed-based*	FC+	BPRS+	putamen	ACC
Han 2019, Sarpal 2016	seed-based	FC-	BPRS+, PANSS+	putamen	insula (cortex)
Han 2019	seed-based	FC+	PANSS-	putamen	insula (cortex)
Sarpal 2016	seed-based	FC-	BPRS+	caudate	ACC
Nelson 2022a	seed-based	FC+	BPRS+	caudate	ACC
Sarpal 2016, Nelson 2022a, Nelson 2022b	seed-based	FC+	BPRS+	caudate	insula (cortex)
Blazer 2022	seed-based	FC-	BPRS	caudate	insula (cortex)
				**striatum**	**DMN**
Nelson 2022a, Sarpal 2016	seed-based	FC+	BPRS+	caudate	PCC
Sarpal 2016	seed-based	FC-	BPRS+	caudate (ventral)	PCC
				**striatum**	**NA**
Blazer 2022, Sarpal 2016	seed-based	FC-	BPRS+	caudate	inferior frontal gyrus

*Task-based: stroop-color-naming task.

**Abbreviations:** FC+: positive correlation between FC and improvement in symptoms: FC-: negative correlations between FC and improvement in symptoms, BPRS: Brief Psychiatric Rating Scale, BPRS+: Brief Psychiatric Rating Scale, positive symptoms, BPRS-: Brief Psychiatric Rating Scale, negative symptoms, PANSS+: Positive and Negative Syndrome Scale, positive symptoms, PANSS -: Positive and Negative Syndrome Scale, negative symptoms, RSN: Resting State Network, VAN: ventral attention network, DMN: default mode network, ACC: anterior cingulate cortex, PCC: posterior cingulate cortex, NA: not applicable.

Furthermore, four studies reported that caudate – insula FC is related to AP-R; three of these reported this connection was positively associated with AP-R (for positive symptoms) (Nelson et al., 2022, Sarpal et al., 2016). In contrast, one study reported this connections is negatively associated with AP-R (for positive symptoms) (Blazer et al., 2022). In addition, two rs studies found FC-dysconnectivity between putamen – insula is negatively associated with AP-R (Han et al., 2020, Sarpal et al., 2016). Finally, one study found dysconnectivity between putamen – insula is positively associated with AP-R (for negative symptoms) (Han et al., 2020).

### 3.4 Q2: FC change related to AP-R

Eighteen studies investigated change in FC between before and after AP treatment in relation to AP-R (Anticevic et al., 2015, Blazer et al., 2022, Cadena et al., 2019, Chopra et al., 2021, Deng et al., 2022, Duan et al., 2020, Hadley et al., 2014, Hadley et al., 2016, Han et al., 2020, Kraguljac et al., 2016, Li et al., 2016, Li et al., 2020, Liu et al., 2022, Sarpal et al., 2015, Shan et al., 2021, Wang et al., 2019, Blessing et al., 2020, Zong et al., 2019). A total of 482 patients were included of which 355 FE patients, 69 chronic patients and 58 patients with unknown state of disease. Table 1b shows an overview of the results for FC change related to AP-R. All studies except one, which used a stroop-color-naming task, used rs fMRI. (Cadena et al., 2019) The interval between baseline and follow-up varied from 1 week to 24 months (median of 8 weeks). Two studies defined AP-R as a priori set percentage (i.e., 30 and 50%) reduction in symptom score (Liu et al., 2022, Deng et al., 2022), the other studies defined AP-R as a percentage of change in clinical symptom (subscale) score. Treatment consisted of risperidone monotherapy (N=7 (Cadena et al., 2019, Duan et al., 2020, Hadley et al., 2014, Hadley et al., 2016, Han et al., 2020, Kraguljac et al., 2016, Zong et al., 2019), olanzapine monotherapy (N=2) (Li et al., 2020, Shan et al., 2021), 1:1 randomization between risperidone and aripiprazole (N=1) (Sarpal et al., 2015), multiple AP-agents, including risperidone (N=5) (Blessing et al., 2020, Wang et al., 2019, Li et al., 2016, Liu et al., 2022, Deng et al., 2022)) or was not reported (N=2) (Anticevic et al., 2015, Chopra et al., 2021). Fourteen studies analyzed fMRI data with a seed-based approach, two studies used ICA (Blessing et al., 2020, Zong et al., 2019), one study used Network-Based Statistics (NBS) (Chopra et al., 2021), one study used also CCA (Blazer et al., 2022) and three studies used a graph theory approach (Wang et al., 2019, Hadley et al., 2016, Liu et al., 2022).

**Fig. 5:**
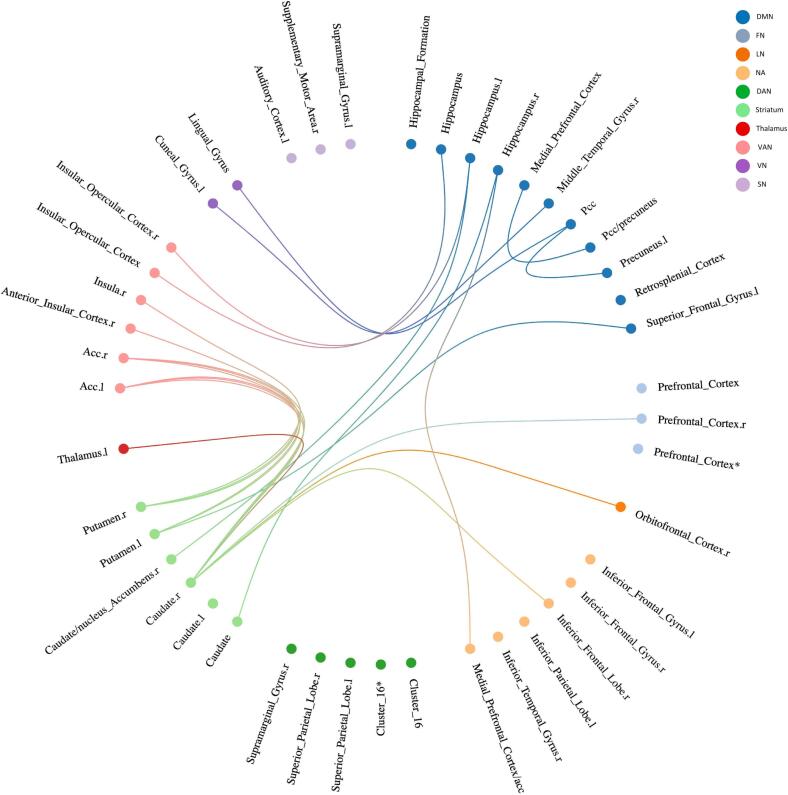
Overview of longitudinal FC increase changed connections related to AP-R. Each significant connection is divided in RSN, shown in corresponding color. If it was not possible to categorize regions of a significant connection in the RSN framework, it was assigned to the NA group. *Connection with itself**Abbreviations:** DMN: default mode network, FN: frontoparietal network, LN: limbic network, NA: Not applicable, SN: sensorimotor network, VAN: ventral attention network, VN: visual network, DAN: dorsal attention network, ACC: anterior cingulate cortex, l: left, PCC: posterior cingulate cortex, r**:** right, VTA: ventral tegmental area.

**Fig. 6:**
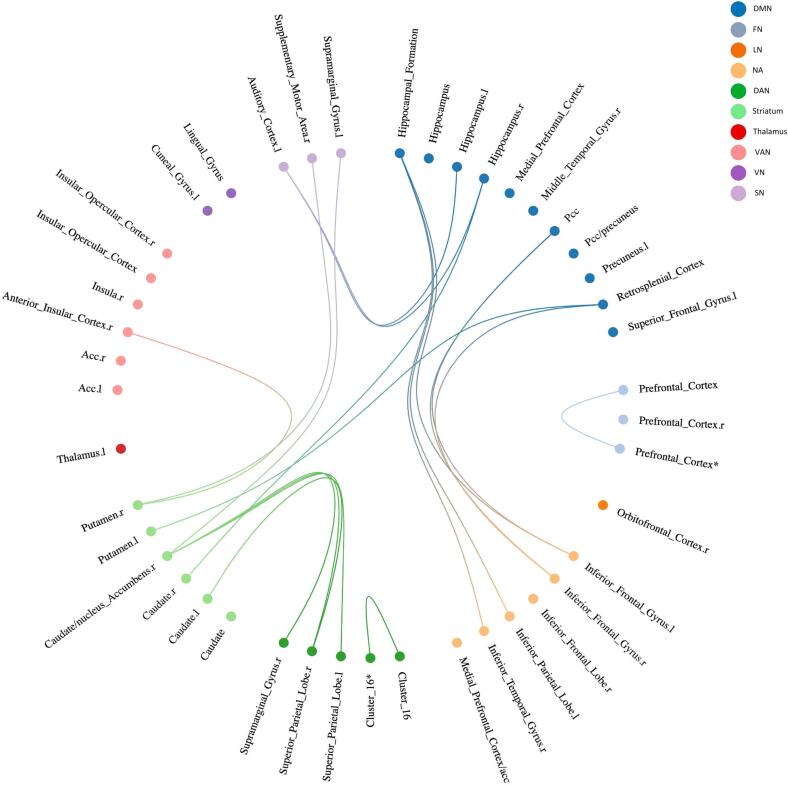
Overview of longitudinal FC decrease changed connections related to AP-R. Each significant connection is divided in RSN, shown in corresponding color. If it was not possible to categorize regions of a significant connection in the RSN framework, it was assigned to the NA group. *Connection with itself**Abbreviations:** DMN: default mode network, FN: frontoparietal network, LN: limbic network, NA: Not applicable, SN: sensorimotor network, VAN: ventral attention network, VN: visual network, DAN: dorsal attention network, ACC: anterior cingulate cortex, l: left, PCC: posterior cingulate cortex, r**:** right, VTA: ventral tegmental area.

In total, 45 unique connections were reported to change with AP-R (Fig. 5, Fig. 6, supplementary file S7) of which three connections were found in more than one study (Table 2b). Most conclusive evidence was found in FC between striatum – VAN and striatum – DMN. These findings are reported in 134 patients, after treatment with risperidone (N=116) or aripiprazole (N=18) with clinical evaluation between 6 and 12 weeks follow-up. Regarding the connections between striatum and VAN: One task-based study (Cadena et al., 2019) and one rs study (Sarpal et al., 2015) found increased FC between putamen and ACC after treatment, which was associated with AP-R (for positive symptoms). One study found increased putamen – insula FC associated with AP-R (positive symptoms) (Sarpal et al., 2015), but another reported decreased FC associated with AP-R (for negative symptoms) (Han et al., 2020). Two studies found that increased rs FC between caudate – hippocampus (a striatum – DMN connection) was associated with AP-R (for positive symptoms) (Kraguljac et al., 2016, Sarpal et al., 2015), and decreased caudate – hippocampus FC was associated with AP-R (for positive symptoms) in one study (Kraguljac et al., 2016).

**Table 2b:** Overview longitudinal changed connections related to AP-R, described by two or more unique studies, categorized by RSN and unique connection (Q2).

**First author, year of publication**	**Analytical approach**	**Direction of FC change between A and B**	**Clinical assessment**	**Region A, corresponding RSN**	**Region B, corresponding RSN**
				**Striatum**	**VAN**
Cadena 2018, Sarpal 2015	seed-based*, seed-based	increase	PANSS+, BPRS+	putamen	ACC
Sarpal 2015	seed-based	increase	BPRS+	putamen	insula
Han 2019	seed-based	decrease	PANSS-	putamen	insula (cortex)
				**Striatum**	**DMN**
Sarpal 2015, Kraguljac 2016	seed-based	increase	BPRS+	caudate	hippocampus
Kraguljac 2016	seed-based	decrease	BPRS+	caudate	hippocampus

*Task-based: Stroop-color-naming task.

**Abbreviations:** FC: functional connectivity, BPRS: Brief Psychiatric Rating Scale, BPRS+: Brief Psychiatric Rating Scale, positive symptoms, BPRS-: Brief Psychiatric Rating Scale, negative symptoms, PANSS+: Positive and Negative Syndrome Scale, positive symptoms, PANSS -: Positive and Negative Syndrome Scale, negative symptoms RSN: Resting State Network, VAN: Ventral attention network, DMN: default mode network, ACC: anterior cingulate cortex.

Graph theoretical studies reported contradicting results. One study found that an increase in global clustering over time was associated with AP-R (negative symptoms), while another found a decrease in global clustering over time that was associated with AP-R (total scores) (Wang et al., 2019, Hadley et al., 2016). An increase of global efficiency was related to AP-R (Hadley et al., 2016). Examining specific brain regions, one study identified a decrease in degree centrality within the striatum (Liu et al., 2022) and another study found a correlation between an increase of network efficiency in two local structures within the DMN and AP-R (Wang et al., 2019). See (Table 2c).

**Table 2c:** Overview results prediction related to AP-R (Q3).

***First author, year of publication***	***Brain region***		***N***	***Treatment outcome***	***Method***	***Input*** ***feature***	***Validation***	***Results***						
	***Region A***	***Region B***						***Accuracy (%)***	***Sensitivity (%)***	***Specificity (%)***	***PPV (%)***	***NPV (%)***	***AUC***	***r-value***
Blessing, 2019	hippocampus (anteromedial)	insular opercular cortex (posterior) (r)	27	BPRS total	RF	Correlation FC	OOB	89.0	NR	NR	NR	NR	0.95	NA
Cao, 2018	superior temporal cortex (b)	cortical regions	43	PANSS total	SVM	Correlation FC	LOO-CV	82.5	88.0	76.9	NR	NR	NR	NA
Cao, 2018	superior temporal cortex (b)	cortical regions	43	PANSS total	SVM	MI	LOO-CV	57.4	84.0	30.8	NR	NR	NR	NA
Li (Ang), 2020	striatum	whole brain	95	PANSS-	SVM	FSA-score	LSO	80.0	79.3	81.5	NR	NR	NR	NA
Li, 2020	ACC	whole brain	32	PANSS-	SVM	GFC	LOO-CV	NR	NR	NR	NR	NR	NR	0.957
Liu, 2022	DMN, striatum, network, cerebellum network	DMN, striatum, network, cerebellum network	38	PANSS total	SVM	Degree centrality	LOO-CV and separate sample	84.2	78.9	89.5	NR	NR	0.925	NA
Nejad, 2012	WM component(r), WM component (b), DMN component	WM component (r), WM component (b), DMN component	14	PANSS-	SVM	Network components*	LOO-CV	78.6	75.0	83.33	NR	NR	NA	NA
Nejad, 2012	WM network components	WM network components	14	PANSS-	SVM	Network components*	LOO-CV	78.6	62.5	100.0	NR	NR	NA	NA
Nejad, 2012	DMN component	DMN component	14	PANSS-	SVM	Network components*	LOO-CV	50.0	62.5	33.33	NR	NR	NA	NA
Nejad, 2012	Frontoparietal WM network component (r)	Frontoparietal WM network component (r)	14	PANSS-	SVM	Network components*	LOO-CV	64.29	62.5	66.67	NR	NR	NA	NA
Nejad, 2012	Frontoparietal WM network component (b)	Frontoparietal WM network component (b)	14	PANSS-	SVM	Network components*	LOO-CV	57.14	50.0	66.67	NR	NR	NA	NA
Sarpal, 2016	striatum	whole brain	41	BPRS total	Hypotheses driven	Correlation FC	Generalizability cohort	75.0	80.0	75.0	76	79	0.78	NA
Shan, 2021	superior/middle MPFC	superior/middle MPFC	20	PANSS total	SVM	VMHC baseline	CV with subsets	NR	NR	NR	NR	NR	NR	0.607
Shan, 2021	superior/middle MPFC	superior/middle MPFC	20	PANSS+	SVM	VMHC baseline	CV with subsets	NR	NR	NR	NR	NR	NR	0.906
Shan,2021	superior/middle MPFC	superior/middle MPFC	20	PANSS-	SVM	VMHC Baseline	CV with subsets	NR	NR	NR	NR	NR	NR	0.774
Shan,2021	superior/middle MPFC	superior/middle MPFC	20	PANSS total, PANSS+,PANSS-, PANSS general	SVM	VMHC alterations	CV with subsets	NR	NR	NR	NR	NR	NR	NS
Zhu, 2018	MTG/inferior temporal gyrus (l)	whole brain	44	PANSS total	SVM	PAS-score	LOO-CV	63.64	72.73	54.55	NR	NR	NR	NA
Zhu, 2018	PCC/precuneus (l)	whole brain	44	PANSS total	SVM	PAS-score	LOO-CV	70.45	63.64	77.27	NR	NR	NR	NA
Zhu, 2018	MTG /inferior temporal gyrus (l), PCC/precuneus (l)	whole brain	44	PANSS total	SVM	PAS-score	LOO-CV	75.00	77.27	72.73	NR	NR	NR	NA
Zhu, 2018	Superior parietal lobe	whole brain	44	PANSS total	SVM	PAS-score	LOO-CV	77.27	77.27	77.27	NR	NR	NR	NA
Zhu, 2018	precentral gyrus/postcentral gyrus	whole brain	44	PANSS total	SVM	PAS-score	LOO-CV	88.64	90.91	86.36	NR	NR	NR	NA
Zhu, 2018	superior parietal lobe, precentral gyrus, postcentral gyrus	whole brain	44	PANSS total	SVM	PAS-score	LOO-CV	93.18	86.36	100.0	NR	NR	NR	NA

* Temporally coherent FC networks form fMRI signals associated with task.

**Aberrations**: b: bilateral, l: left, r: right, WM: working memory, DMN: defaultmode network, BPRS: Brief Psychiatric Rating Scale, BPRS+: Brief Psychiatric Rating Scale, positive symptoms, BPRS-: Brief Psychiatric Rating Scale, negative symptoms, PANSS+: Positive and Negative Syndrome Scale, positive symptoms, PANSS -: Positive and Negative Syndrome Scale, negative symptoms RF: Random forest, SVM: support vector machine, FC: functional connectivity, PAS-score: parameter of asymmetry-score MI: mutual information, FSA-score: functional striatal–abnormalities - score OOB:, LOO-CV: leave-one-out cross validation, LSO: leave-site-out, NR: not reported, NA: not applicable, PPV: positive predictive value, NPV: negative predictive value, AUC: area under the curve, VMHC: voxel-wise homotopic.

### 3.5 Q3: FC predicting AP-R

Nine studies investigated the prediction of AP-R based on fMRI-FC (Li et al., 2020, Li et al., 2020, Liu et al., 2022, Sarpal et al., 2016, Shan et al., 2021, Zhu et al., 2018, Blessing et al., 2020, Cao et al., 2018, Nejad et al., 2013). Eight studies used rs fMRI, and one study used an N-back task (Nejad et al., 2013). A total of 310 patients were included, of which 157 FE patients, 95 patients with an unknown state of disease, and two studies included both (N = 58) (Liu et al., 2022, Shan et al., 2021). Table 1c shows an overview of the results. Both whole brain and ROI approaches were reported.

The interval between baseline and follow-up varied in the studies from 8 weeks to 7 months (median of 8 weeks). Five studies defined AP-R as a percentage of change in clinical symptom score (Sarpal et al., 2016, Zhu et al., 2018, Blessing et al., 2020, Nejad et al., 2013). Three studies defined AP-R as a prior set percentage (i.e., 20% 35%, 50%) symptom reduction (Li et al., 2020, Sarpal et al., 2016, Cao et al., 2018). Four studies aimed to predict AP-R with risperidone (one study (Cao et al., 2018), olanzapine (three studies (Li et al., 2020, Shan et al., 2021, Zhu et al., 2018) or quetiapine (one study (Nejad et al., 20132013). One study applied 1:1 randomization between risperidone and aripiprazole (Sarpal et al., 2016); and patients were treated with variable AP in three studies (Li et al., 2020, Liu et al., 2022, Blessing et al., 2020). Regarding fMRI methodology, seven studies used a seed-based approach and two implemented ICA (Blessing et al., 2020, Nejad et al., 2013); seven out of nine studies used a support vector machine (SVM) to classify their data (Anticevic et al., 2015, Kraguljac et al., 2016, Li et al., 2020, Liu et al., 2022, Shan et al., 2021, Zhang et al., 2019, Zong et al., 2019). One study used a random forest (RF) model (Blessing et al., 2020) and one study tested the predictive value of a hypothesis-driven index (Sarpal et al., 2016). Only three studies validated their results in an independent sample; by using an independent cohort (Sarpal et al., 2016), leave-one-site-out (LOSO) (Li et al., 2020) or they had used use a separate sample approach (Liu et al., 2022). The validation of results was most often performed by leave-one-out cross validation (LOOCV) approach (Li et al., 2020, Cao et al., 2018, Nejad et al., 2013, Zhu et al., 2018). The accuracy of fMRI-FC based predictors of AP-R differed between 57.4% and 93.2%, with 54.6%-100% specificity and 50.0%-90.9% sensitivity. The results for the studies including an independent sample validation approach varied between 75% and 84.2%. Two of the studies including an independent test cohort investigated the FC between striatum and whole brain (N = 41 and N = 95 patients) resulting in 80% and 75% accuracy respectively (Sarpal et al., 2016, Li et al., 2020). Patients were treated with variable AP (mean olanzapine equivalent dose 15.5 mg) and clinical evaluations took place after 6 to 12 weeks. The other study that included a validation dataset investigated degree centrality of different brain regions including the striatum (Liu et al., 2022). The six studies without a validation sample investigated AP-R prediction based on FC between various brain regions (Cao et al., 2018, Li et al., 2020, Shan et al., 2021, Zhu et al., 2018, Blessing et al., 2020, Nejad et al., 2013).

## 4 Discussion

Our current systematic review of the literature on fMRI-FC in relation to AP-R for psychosis showed that this is a relatively new field where methodology is still rapidly evolving. We found dysconnectivity between striatum – VAN and striatum -DMN at baseline, with varying patterns of change over time. Key regions of interest included the putamen and caudate nucleus in the striatum, as well as the insula and ACC in the VAN. Notably, both hypoconnectivity and hyperconnectivity were observed, with subsequent increases or decreases in FC over time reported. Those divers FC alterations over time weakens the evidence for the hypothesis that a dysconnectivity at baseline is normalized by treatment with AP. Prediction studies reported high accuracies for AP-R, but they were heterogeneous in the specific connections used as input for the prediction models. The methodological quality of studies was generally good, but mainly based on relatively small sample sizes. We found high variety of methodological approaches (e.g., different fMRI paradigms, analysis pipelines), and we identified no studies that reported attempts to replicate or falsify previous work.

The striatum emerged as the most extensively studied brain area, with FC-dysconnectivity of the striatum serving as an indicator of potential AP-R impact in twelve out of seventeen baseline studies. However, the findings of the literature should be interpreted with caution. Still, only seven out of these twelve studies reported significant striatal connections related to AP-R. Similarly, FC changes in the striatum associated with AP-R were investigated in eleven out of nineteen studies examining changes over time, but only five of these studies found significant striatal connections.

The findings of this review can be integrated with the dopamine hypothesis of schizophrenia. This theory states that disrupted dopaminergic transmission in striatum is a key pathophysiological characteristic of psychosis, and a key mechanism of AP-treatments involves their binding to dopamine receptors (Howes and Kapur, 2009, Friston and Frith, 1995, Friston et al., 2016). Moreover, it has been suggested that inter individual variation in striatal connectivity is dysfunctional in schizophrenia (Martino et al., 20182018, McCutcheon et al., 2019).

### 4.1 Methodological issues and future directions

Factors related to clinical study design, fMRI acquisition, fMRI processing, and machine learning methodology limit translation to the clinic in this field. The reviewed studies differed in their views on minimum required AP-treatment doses and duration to establish AP-R. It has been suggested that AP-NR should be evaluated after at least ten weeks of AP-treatment (Kahn et al., 2018), which was only the case for twelve out of 28 studies. Furthermore, twelve studies included chronic schizophrenic patients (Nelson et al., 2022, Cadena et al., 2019, Doucet et al., 2020, Hadley et al., 2014, Kraguljac et al., 2016, Kraguljac et al., 2016, Zhang et al., 2019, Hadley et al., 2016, Liu et al., 2022, Shan et al., 2021, Anticevic et al., 2015, Blazer et al., 2022). The Treatment Response and Reactance in Psychosis (TRRIP) workgroup advises a dose of 20 mg of olanzapine in case of treatment resistant psychosis (Howes et al., 2017), which was only the case in one study (Shan et al., 2021). Overall, it is difficult to assess an optimal dose for patients that do not respond to AP, suggesting that AP-NR may be inadequately assessed (van Dellen, 2023).

In addition, the majority of the studies solely focus on AP as the treatment intervention, without consideration of placebo-, Hawthorne- and natural disease course effects. A notable exception is the study by Chopra et al. (Chopra et al., 2021), who conducted a placebo-controlled triple-blind investigation in which psychotherapy served as the intervention for the placebo group. Consequently, it remains unclear whether AP-R observed in the remaining studies is solely attributable to the administration of AP or influenced by other interventions.

Concerning fMRI acquisition, previous studies have shown that many non-neural factors may influence the variation in fMRI signals and, consequently, the reliability of the intra- and inter-individual FC (Hong et al., 2020, Birn et al., 2008). One example is scanning duration. Nine minutes of scanning may be considered a minimum to show reliable FC estimates, but the scanning time was shorter in all but one of the included studies (Blazer et al., 2022, Birn et al., 2008). Other factors limiting comparability between studies are a task versus rs scanning conditions, and eyes open versus closed measurements (Patriat et al., 2013). The field would benefit from consensus on scanning conditions that maximize power for AP-R effects.

A third factor is heterogeneity in fMRI signal analysis pipelines. FC features with higher reliability have higher accuracy in prediction studies, while fMRI signals can be disrupted by particular patient characteristics (e.g., hemodynamics, arterial CO2 concentration, blood pressure, cerebral/cerebellar autoregulation) (Birn et al., 2008, Birn et al., 2006, Wang et al., 2006, Wise et al., 2004). In the context of AP-R, it must be considered that these disturbances vary between individuals and may change within individuals between measurements. Head movement during scanning is a non-neural-factor which needs consideration as this is known to interact with the psychotic disease state (Van Dijk et al., 2012, Power et al., 2012), while AP-R may be associated with increased (or decreased) motion (Satterthwaite et al., 2012). Noise removal techniques may provide more accurate measures, although the impact of these corrections is not straightforward because artefacts may be related to the signals of interest; the impact of variation in data scrubbing techniques and optimal standardization is still debated. As a result, data acquisition and processing standards varied considerably between studies included in this review. For example, four studies used global signal regression for physiological motion correction, which is a risk of discarding global neural information (Power et al., 2014, Murphy and Fox, 2017). Recent work explored a set of benchmark parameters for the use of FC as a tool in the domain of biomarker discovery including: using a linear dimensionality reduction algorithm, utilizing the gradients that explain a greater amount of the variance of the data and extracting gradients using more conservatively threshold FC matrices (Hong et al., 2020). Future work in this direction and exploration of large-sample test–retest neuroimaging datasets, will be valuable in biomarker development and may impact the interpretation of findings summarized in this review.

Fourth, several machine learning aspects must be noted in prediction studies aiming to make individual-level clinical predictions of AP-R (Janssen et al., 2018). The sample sizes of most of the studies were small, which might cause overfitting (Janssen et al., 2018, Bzdok and Meyer-Lindenberg, 2018). The performance of these algorithms should be evaluated by validation procedures (Janssen et al., 2018, Bzdok and Meyer-Lindenberg, 2018). We found that different machine learning algorithms were used with different validation methods. The optimal validation method uses a separate training and test-set, to verify results in an independent sample (Haynes, 2015), which was only done in two studies (Sarpal et al., 2016, Li et al., 2020). In addition, future research should provide a clinical generalizable predictor, for example, to populations with the same mental disorder but different comorbidity, and across countries and cultures (Coutts et al., 2023). In summary, optimal handling of fMRI data is a challenging issue and various processing choices will be critical.

### 4.2 Strengths and limitations

This review included studies that addressed fMRI-FC in different types of longitudinal designs (baseline, change and prediction) with different types of fMRI-FC analyses (seed-based, CCA, ICA, NBS graph theory). To distinguish between the quality of different studies, we critically assessed all studies and developed ROB based on previous literature. While we have accounted for various factors in our clinical and fMRI methodological evaluations, there are still some limitations which may impact the quality of evidence.

We developed our ROB tool based on prior literature on biomarker identification (Lueken et al., 2016). Consequently, we omitted certain characteristics which could be influential. In our study, we incorporated every study, regardless of its ROB-score. Regarding methodological aspects, there appears to be insufficient consensus in the field for a robust ROB assessment. We therefore based our methodological ROB assessment on the state of art methodological studies (van Montfort et al., 2019). As a result, certain aspects, such as thresholding, were not accounted in our assessment, even though they can significantly influence the reliability of the findings (Lieberman and Cunningham, 2009). Another limitation is that connections were categorized according to literature on whole-brain network parcellation. We should be cautious as this exploratory analysis could only approximately define RSNs, since FC-seeds are not permanently fixed within the 7-large scale networks. Moreover, publication bias might have affected our results towards the involvement of the striatum and DMN, as these regions were most often studied. We included studies on FE patients as well as chronic schizophrenia patients in our review, where FE logically is more responsive to treatment than more chronic patients, and therefore might be difficult to compare. In line with this, we compared AP-R from different AP, including clozapine, which was evaluated by two studies (Li et al., 2016, Blazer et al., 2022). However clozapine is known to be more effective than other AP (Leucht et al., 2013), which makes it less reliable to compare these studies. Last, in our review, we evaluated methods that have been implemented for an extended period of time. Other methods, such as dynamic FC (Zhang et al., 2021, Kottaram et al., 2020), were excluded from our review, given their limited current application in the field. However, if this area further develops, it is essential that future research will consider these methods as well (Zhang et al., 2021, Kottaram et al., 2020). By limiting our review to FC, our aim was to ensure that the examined studies were directly comparable, enhancing the consistency and reliability of our analyses and conclusions. This was challenging due to the heterogeneity of the studies.

## 5 Conclusion

In conclusion, our systematic review shows preliminary evidence that fMRI-FC analysis of especially striatal-VAN and striatal-DMN coupling is worth further study as it holds some promise as a potential predictor or biomarker of AP-R. The current fMRI-FC literature on AP-R is however hampered by heterogeneity of methodological approaches and small sample sizes. Harmonization and optimalization of fMRI connectivity analysis is needed before clinical applications can be expected. The use of more stringent study methodology, standardized analysis pipelines, consistent outcome measures and consolidation of large datasets from multicenter studies is required before fMRI-FC analysis could be of value in clinical practice for a more tailored choice of AP-treatment.

## Declaration of Competing Interest

The authors declare that they have no known competing financial interests or personal relationships that could have appeared to influence the work reported in this paper.

## Data Availability

No data was used for the research described in the article.
