# Supplementary material for: fMRI connectivity as a biomarker of antipsychotic treatment response: A systematic review
Source: Neuroimage Clin. 2023 Sep 23;40:103515. doi: 10.1016/j.nicl.2023.103515 (PMC10568423; doi:10.1016/j.nicl.2023.103515)
Supplement: Supplementary data 1 [file mmc1.docx]

**fMRI connectivity as a biomarker of antipsychotic treatment response: a systematic review – Supplementary files**

**Content:**

**Supplementary file S1 -** Search strategies

Table S1

Table S2

**Supplementary file S2** - Calculation Defined Daily Dose

Table S3

**Supplementary file S3 -** Selection process

Figure S1

**Supplementary file S4** - Risk of Bias assessment

Table S4

**Supplementary file S5 -** Baseline FC related to AP-R

Table S5, Table S6

**Supplementary file S6** – Clusters

Table S7

**Supplementary file S7 –** Longitudinal FC change related to AP-R

Table S8, Table S9

**Supplementary file S1 -** Search strategies

**Supplementary Table S1 -** Pubmed search terms (A + B + C + D)

| Part A | Part B | Part C | Part D |
| --- | --- | --- | --- |
| (((((("Schizophrenia"[Mesh]) OR (schizophren*[Title/Abstract])) OR (schizoaffectiv*[Title/Abstract])) OR (psychos*[Title/Abstract])) OR ("psychotic disorder*"[Title/Abstract])) OR ("Psychotic Disorders"[Mesh])) | ((((Neuroleptic*[Title/Abstract]) OR ("Major Tranquilizer*"[Title/Abstract])) OR (antipsychotic*[Title/Abstract])) OR ("Antipsychotic Agents"[Mesh])) | (((((((((((((((((((((MR[Title/Abstract]) OR (NMR[Title/Abstract])) OR (MRI[Title/Abstract])) OR ("Magnetic Resonance Imaging"[Title/Abstract])) OR ("Magnetic Resonance Imaging"[Mesh])) OR ("Functional Magnetic Resonance Imaging"[Title/Abstract])) OR ("Functional MRI*"[Title/Abstract])) OR (fMRI[Title/Abstract])) OR (“Resting-state functional connectivity”[Title/Abstract])) OR ("functional cerebral localization*"[Title/Abstract])) OR ("brain electrical activity mapping"[Title/Abstract])) OR ("brain mapping"[Title/Abstract])) OR ("Brain Mapping"[Mesh])) OR ("graph analysis"[Title/Abstract])) OR (network*[Title/Abstract])) OR (connectivity[Title/Abstract])) OR ("Connectome"[Mesh])) OR ("connectome*"[Title/Abstract])) OR (“resting-state”[Title/Abstract])) OR (“resting state”[Title/Abstract])) OR (“neuroimaging”[Title/Abstract])))) | ((((((((((((((((((((((((((((“Patient-Relevant Outcome*”[Title/Abstract]) OR ("Rehabilitation Outcome*"[Title/Abstract])) OR (“treatment resistanc*”[Title/Abstract])) OR ("Treatment Efficacy"[Title/Abstract])) OR ("Clinical Efficacy"[Title/Abstract])) OR ("Treatment Outcome"[Mesh])) OR ((((((“Patient-Relevant Outcome*”[Title/Abstract]) OR ("Rehabilitation Outcome*"[Title/Abstract])) OR (“treatment resistanc*”[Title/Abstract])) OR ("Treatment Efficacy"[Title/Abstract])) OR ("Clinical Efficacy"[Title/Abstract])) OR ("Treatment Outcome"[Mesh]))) OR ("Sensitivity and Specificity"[MeSH])) OR (Sensitivity[Title/Abstract])) OR (Specificity[Title/Abstract])) OR ("Predictive Value of Tests"[Mesh])) OR (predict*[Title/Abstract])) OR (response*[Title/Abstract])) OR ("biomarker*"[Title/Abstract])) OR ("Clinical marker*"[Title/Abstract])) OR ("biologic marker*"[Title/Abstract])) OR ("Biomarkers"[Mesh])) ) OR ("clinical outcome*"[Title/Abstract])) OR (remission[Title/Abstract])) OR (responsiveness[Title/Abstract])) OR (Responder*[Title/Abstract])) OR ("nonresponse"[Title/Abstract])) OR (non-responder*[Title/Abstract])) OR (effectiveness[Title/Abstract])) |

**Supplementary Table S2 -** EMBASE search terms (A + B + C + D)

| **Part A** | **Part B** | **Part C** | **Part D** |
| --- | --- | --- | --- |
| 'schizophrenia spectrum disorder'/exp OR 'schizophrenia spectrum disorder' OR schizophren*:ti,ab,kw OR schizoaffectiv*:ti,ab,kw OR psychos*:ti,ab,kw OR psychot*:ti,ab,kw OR 'psychotic disorders':ti,ab,kw OR 'psychosis'/exp | “neuroleptic:ti,ab,kw OR 'major tranquilizers':ti,ab,kw OR antipsychotic*:ti,ab,kw OR 'neuroleptic agent'/exp) | 'magnetization transfer contrast imaging':ti,ab,kw OR mr:ti,ab,kw OR nmr:ti,ab,kw OR mri:ti,ab,kw OR 'magnetic resonance imaging':ti,ab,kw OR 'nuclear magnetic resonance imaging'/exp OR 'functional magnetic resonance imaging':ti,ab,kw OR 'functional mri*':ti,ab,kw OR fmri:ti,ab,kw OR 'resting-state functional connectivity':ti,ab,kw OR 'magneto encephalogram':ti,ab,kw OR meg:ti,ab,kw OR 'electroencephalography'/exp OR electroencephalograph*:ti,ab,kw OR eeg:ti,ab,kw OR electroencephalogram*:ti,ab,kw OR 'functional cerebral localization*':ti,ab,kw OR 'brain electrical activity mapping':ti,ab,kw OR 'brain mapping':ti,ab,kw OR 'brain mapping'/exp OR 'graph analysis':ti,ab,kw OR network*:ti,ab,kw OR connectivity:ti,ab,kw OR 'connectome'/exp OR 'connectome*':ti,ab,kw OR 'resting-state':ti,ab,kw OR 'resting state':ti,ab,kw OR 'electrocorticograph*':ti,ab,kw OR 'magnetoencephalography':ti,ab,kw OR 'neuroimaging':ti,ab,kw OR qeeg:ti,ab,kw | 'patient-relevant outcome*':ti,ab,kw OR 'rehabilitation outcome*':ti,ab,kw OR 'treatment resistanc*':ti,ab,kw OR 'treatment efficacy':ti,ab,kw OR 'clinical efficacy':ti,ab,kw OR 'treatment outcome'/exp OR 'treatment effectiveness':ti,ab,kw OR 'treatment outcome':ti,ab,kw OR 'sensitivity and specificity'/exp OR sensitivity:ti,ab,kw OR specificity:ti,ab,kw OR 'predictive value'/exp OR 'predictive value of tests':ti,ab,kw OR predict*:ti,ab,kw OR response*:ti,ab,kw OR 'biomarker*':ti,ab,kw OR 'clinical marker*':ti,ab,kw OR 'biologic marker*':ti,ab,kw OR 'biological marker'/exp OR 'clinical outcome*':ti,ab,kw OR 'non-response':ti,ab,kw OR remission:ti,ab,kw OR responsiveness:ti,ab,kw OR responder*:ti,ab,kw OR 'nonresponse':ti,ab,kw OR 'non responder*':ti,ab,kw OR nonresponder*:ti,ab,kw OR effectiveness:ti,ab,kw |

**Supplementary file S2 - Calculation Defined Daily Dose**

We calculated the daily defined dose (DDD) with dose equivalents conform the World Health organization Daily Defined Dose method. (Leucht et al., 2016; *World Health Organization Collaborating Centre for Drug Statistics Methodology: ATC/DDD Methodology and ATC/DDD Index 2020.*, n.d.)All medication was recalculated to olanzapine. If dose ranges were given of individual participants, we used the highest dose per participant to calculate the mean dose. In some cases, doses of chlorpromazine equivalents (CPZE) were given instead of doses of the original medication. We then calculated the DDD of chlorpromazine to olanzapine.

**Supplementary file S3 - Selection process**

**
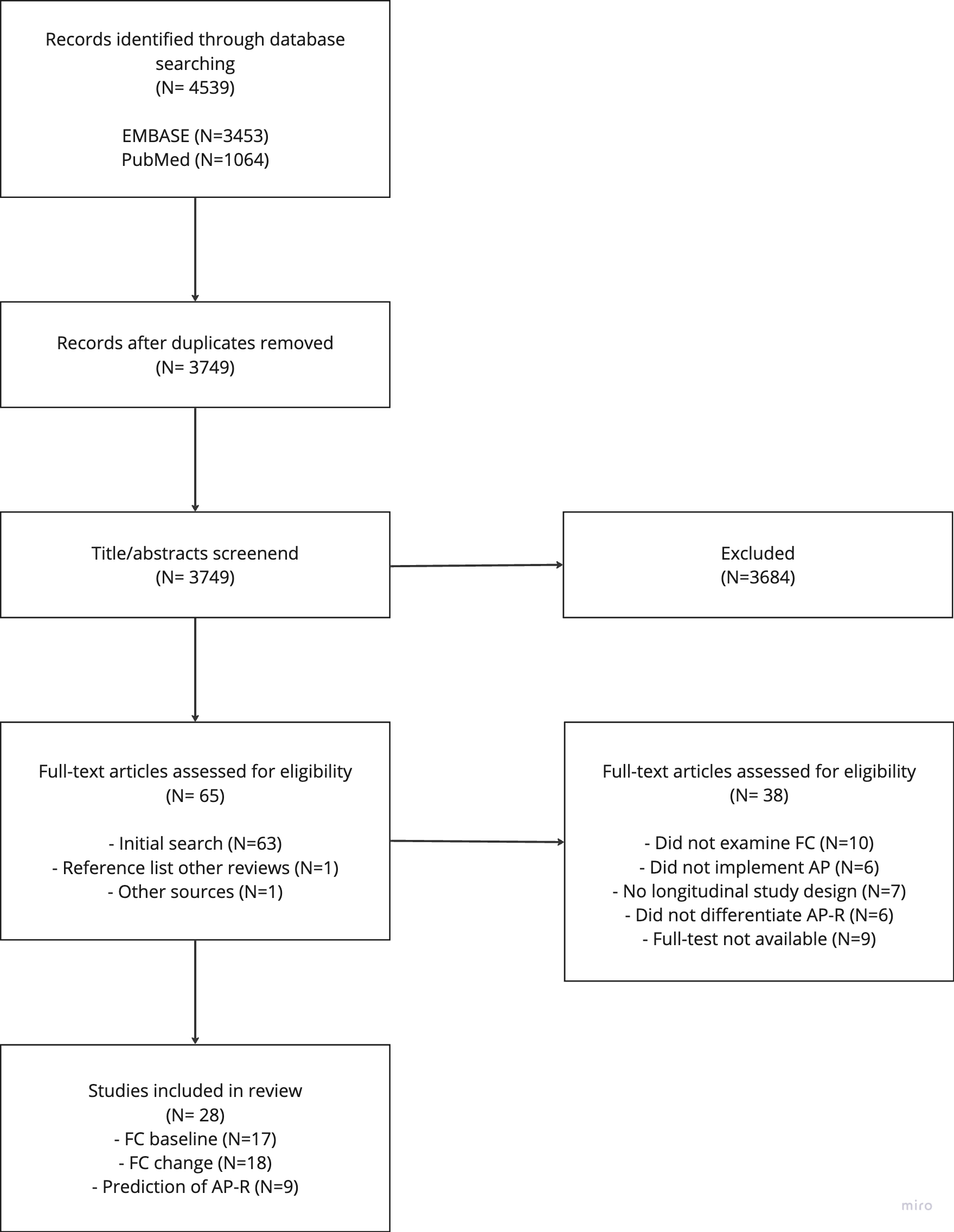
**

**Supplementary** **figure S1 -** Flowchart of the systematic selection process of included articles.

**Abbreviations:** FC: Functional connectivity, AP: Antipsychotic medication, AP-R: Antipsychotic treatment response, AP-NR: Antipsychotic treatment non-response

**Supplementary file S4 - Risk of Bias assessment**

**Supplementary Table S3 -** Risk of bias assessment tool

**Abbreviations:** DDD: Defined daily dose

| **Clinical Assessment (0-18 points)** | | | |
| --- | --- | --- | --- |
|  | **2 points** | **1 point** | **0 points** |
| **Sample (0-4 points)** | | | |
| Independent sample | Yes | Partly | No |
| Sample size* | Large | Medium | Small |
| **Confounder control (0-8 points)** | | | |
| Exclusion criteria | Reported |  | Not reported |
| Concomitant medication | Reported, No | Reported, Yes | Not reported |
| Medication at baseline | Reported, Naive | Reported, free interval or partly naive | Medicated or Not reported |
| Dosage | Adequate (>10 mg of DDD) | Sometimes below therapeutic threshold | Below therapeutic threshold or not reported |
| **Outcome measures (0-2 points)** | | | |
| Statistical control for confounding if applicable | Yes |  | No |
| Primary outcome defined | Yes |  | No |
| **Excellent quality: 16- 18 points**  **Good quality: 10 – 16 points**  **Moderate quality: <10 points** | | | |

*L: N>24 M:N=15-24 S: N<15

| **fMRI methodology (0-5 points)** | | | |
| --- | --- | --- | --- |
|  | 2 points | 1 point | 0 points |
| Motion correction | Spike Regression/scrubbing/group differences evaluated | Reported | No or not reported |
| Duration of scanning | Reported, duration >9 minutes | Reported, duration <9 minutes | Not reported |
| Condition of eyes |  | Reported | Not reported |
| **Excellent quality: 5 points**  **Good quality: 3 or 4 points**  **Moderate quality: <3 points** | | | |

**Supplementary table S4** - Scoring Risk of bias

| **Study Number, First author, year of publication** | **Clinical parameters** | | | | | | | | | **fMRI methodological parameters** | | | |
| --- | --- | --- | --- | --- | --- | --- | --- | --- | --- | --- | --- | --- | --- |
|  | Independent sample | Sample size | Exclusion criteria | Concomitant Medication | Medication at baseline | Dosage | Statistical control for confounding | Primary outcome defined | Total points Clinical | Motion correction | Duration of scanning | Eye condition | Total Points fMRI |
| 1. Anticevic, 2015 | 2 | 2 | 2 | 0 | 2 | 0 | 2 | 0 | 10 | 2 | 1 | 0 | 3 |
| 1. Blessing, 2019 | 2 | 2 | 0 | 0 | 2 | 2 | 2 | 2 | 12 | 1 | 0 | 1 | 2 |
| 1. Blazer, 2022 | 2 | 1 | 2 | 1 | 0 | 1 | 2 | 2 | 11 | 2 | 2 | 1 | 5 |
| 1. Cadena, 2018 | 0 | 1 | 2 | 1 | 1 | 0 | 2 | 2 | 9 | 2 | 0 | 1 | 3 |
| 1. Cao, 2018 | 2 | 2 | 2 | 1 | 2 | 0 | 2 | 2 | 13 | 1 | 1 | 0 | 2 |
| 1. Chopra, 2021 | 2 | 2 | 0 | 1 | 2 | 1 | 2 | 2 | 12 | 1 | 0 | 1 | 2 |
| 1. Doucet, 2018 | 2 | 2 | 2 | 0 | 1 | 0 | 2 | 2 | 11 | 2 | 1 | 1 | 4 |
| 1. Deng, 2022 | 2 | 2 | 2 | 0 | 2 | 1 | 2 | 0 | 11 | 2 | 0 | 1 | 3 |
| 1. Duan, 2020 | 2 | 2 | 0 | 1 | 2 | 0 | 2 | 2 | 11 | 2 | 1 | 1 | 4 |
| 1. Hadley, 2013 | 0 | 2 | 2 | 0 | 1 | 0 | 2 | 2 | 9 | 2 | 1 | 1 | 4 |
| 1. Hadley, 2016 | 1 | 2 | 2 | 1 | 1 | 1 | 2 | 2 | 12 | 2 | 1 | 1 | 4 |
| 1. Han, 2020 | 2 | 2 | 2 | 2 | 2 | 0 | 2 | 2 | 14 | 2 | 0 | 1 | 3 |
| 1. Kraguljac, 2015 | 0 | 2 | 2 | 1 | 1 | 0 | 2 | 2 | 10 | 2 | 1 | 1 | 4 |
| 1. Kraguljac, 2016 | 0 | 2 | 2 | 1 | 1 | 0 | 2 | 2 | 10 | 2 | 1 | 1 | 4 |
| 1. Li (Ang) , 2020 | 2 | 2 | 2 | 0 | 0 | 2 | 2 | 2 | 12 | 1 | 1 | 1 | 3 |
| 1. Li, 2020 | 2 | 2 | 2 | 0 | 2 | 2 | 2 | 2 | 14 | 2 | 1 | 1 | 4 |
| 1. Li, 2016 | 2 | 1 | 2 | 0 | 2 | 0 | 2 | 0 | 9 | 2 | 1 | 1 | 4 |
| 1. Liu, 2022 | 2 | 2 | 2 | 0 | 0 | 1 | 2 | 2 | 11 | 2 | 0 | 1 | 3 |
| 1. Maximo, 2021 | 1 | 2 | 2 | 1 | 1 | 1 | 2 | 2 | 12 | 2 | 0 | 1 | 3 |
| 1. Nelson, 2022 * | 1 | 2 | 2 | 1 | 1 | 1 | 2 | 2 | 12 | 2 | 0 | 1 | 3 |
| 1. Nejad, 2012 | 2 | 0 | 2 | 1 | 2 | 2 | 2 | 2 | 13 | 0 | 0 | 0 | 0 |
| 1. Sarpal, 2014 | 0 | 1 | 2 | 1 | 1 | 1 | 2 | 2 | 10 | 1 | 1 | 1 | 3 |
| 1. Sarpal, 2016 | 1 | 2 | 2 | 1 | 1 | 1 | 2 | 2 | 12 | 1 | 1 | 1 | 3 |
| 1. Shan, 2021 | 2 | 1 | 2 | 0 | 1 | 2 | 2 | 2 | 12 | 2 | 1 | 1 | 4 |
| 1. Wang, 2018 | 2 | 2 | 2 | 0 | 1 | 1 | 2 | 2 | 12 | 1 | 0 | 1 | 2 |
| 1. Zhang, 2019 | 2 | 2 | 2 | 0 | 2 | 0 | 2 | 0 | 10 | 1 | 0 | 1 | 2 |
| 1. Zhu, 2018 | 2 | 2 | 2 | 0 | 2 | 2 | 2 | 2 | 14 | 1 | 0 | 1 | 2 |
| 1. Zong, 2018 | 2 | 2 | 2 | 1 | 2 | 1 | 2 | 0 | 12 | 2 | 0 | 1 | 3 |

**Supplementary S5: Baseline connections related to AP-R**

**Supplementary Table S5 -** Baseline Graph theory

**Abbreviations:** NS: No significance, rs: resting-state, FDR: False discovery rate, PANSS: Positive and Negative Symptom Scale

| **First Author, year of publication** | **Network characteristic** | **Correlation** | **Clinical assessment** | **R value*** | **P-value**** |
| --- | --- | --- | --- | --- | --- |
| Wang, 2019 | Clustering coefficient (global) | NS | PANSS general | -0.096 | 0.746 |
| Wang, 2019 | Clustering coefficient (global) | NS | PANSS+ | -0.069 | 0.746 |
| Wang, 2019 | Clustering coefficient (global) | NS | PANSS- | 0.382 | 0.124 |
| Wang, 2019 | Clustering coefficient (global) | NS | PANSS total | 0.059 | 0.746 |

*r was calculated using partial correlation

**p was corrected for multiple correlations using FDR

**Supplementary Table S6** - Baseline connections related to AP-R

**Abbreviations:** CCA: canonical correlation analysis, FC+: positive correlations between signals FC-: anticorrelated signals, BPRS: Brief Psychiatric Rating Scale, PANSS: Positive and Negative Syndrome Scale, RSN: Resting State Network, VAN: Ventral attention network, L: left, R: Right, DMN: Default mode network, SN: Sensorymotornetwork, VN: Visual network. Structures: ACC: Anterior cingulate cortex, PCC: Posterior Cingulate cortex

| First author, year of publication | Analytical approach | Region A, corresponding RSN | Region B | Correlation with symptom score | Clinical assesment |
| --- | --- | --- | --- | --- | --- |
|  |  | **DMN** | **DMN** | **Unique connections: 10** | **total connection: 12** |
| Blessing, 2019 | ICA | Superior frontal gyrus. R | Hippocampus (anteromedial). L | FC+ | BPRS+ |
| Doucet, 2018 | CCA | Precuneus (dorsal central) | Hippocampus | FC+ | BPRS+ |
| Doucet, 2018 | CCA | Precuneus (dorsal central) | Hippocampus, Amygdala, Temporal Pole, Middle temporal gyrus, Parahippocampal gyrus, Fusiform gyrus | FC+ | BPRS+ |
| Doucet, 2018 | CCA | Precuneus (dorsal central) | Hippocampus, Amygdala, Temporal Pole, Middle temporal gyrus, Parahippocampal gyrus, Fusiform gyrus | FC+ | BPRS+ |
| Doucet, 2018 | CCA | Hippocampus, Amygdala, Temporal Pole, Middle temporal gyrus, Parahippocampal gyrus, Fusiform gyrus | Precuneus (Dorsal posterior), Superior parietal lobe | FC+ | BPRS+ |
| Doucet, 2018 | CCA | Precuneus (Dorsal posterior) | Precuneus (dorsal posterior) Superior parietal lobe | FC- | BPRS+ |
| Doucet, 2018 | CCA | Precuneus (Dorsal central) | Precuneus (Anterior), Posterior Cingulate Cortex | FC- | BPRS+ |
| Doucet, 2018 | CCA | Medial prefrontal cortex, Angular gyri, Inferior temporalgyrus, inferior frontal gyrus | Medial prefrontal cortex, Angular gyri, Inferior temporalgyrus, inferior frontal gyrus | FC- | BPRS+ |
| Zong, 2018 | ICA | PCC/Precuneus.L | Angular gyrus.R | FC- | PANSS- |
| Zong, 2018 | ICA | PCC/Precuneus | Angular gyrus.R | FC- | PANSS- |
| Deng, 2022 | Seed-based | PCC | Precuneus.L | FC- | SAPS |
| Maximo, 2021 | Seed-based | PCC/Precuneus, Medial prefrontal, Angular gyrus | PCC/Precuneus, Medial prefrontal, Angular gyrus | NS | BPRS+ |
|  |  | **Striatum** | **SN** | **Unique connections: 10** | **total connection: 15** |
| Sarpal, 2016 | Seed-based | Caudate (ventral).L | Supramarginal gyrus | FC+ | BPRS+ |
| Sarpal, 2016 | Seed-based | Caudate (ventral).R | Supramarginal gyrus | FC- | BPRS+ |
| Sarpal, 2016 | Seed-based | Caudate (ventral).R | Supramarginal gyrus | FC- | BPRS+ |
| Sarpal, 2016 | Seed-based | Caudate (ventral).R | Planum temporale | FC- | BPRS+ |
| Sarpal, 2016 | Seed-based | Caudate (Dorsal).L | Planum temporale | FC+ | BPRS+ |
| Sarpal, 2016 | Seed-based | Caudate (ventral).R | Supramarginal gyrus | FC- | BPRS+ |
| Sarpal, 2016 | Seed-based | Caudate (Dorsal).R | Precentral gryus | FC- | BPRS+ |
| Sarpal, 2016 | Seed-based | Caudate (Dorsal).R | Precentral gyrus | FC- | BPRS+ |
| Nelson, 2022b | Seed-based | Caudate.B | Sensory motor area | FC+ | BPRS+ |
| Sarpal, 2016 | Seed-based | Putamen (ventral rostal).R | Supramarginal gyrus.R | FC- | BPRS+ |
| Sarpal, 2016 | Seed-based | Putamen (ventral rostal).R | Postcentral gyrus | FC- | BPRS+ |
| Sarpal, 2016 | Seed-based | Putamen (dorsal rostral).R | Precentral gyrus | FC- | BPRS+ |
| Sarpal, 2016 | Seed-based | Putamen (dorsal caudal).L | planum temporale | FC- | BPRS+ |
| Sarpal, 2016 | Seed-based | Nucleus accumbens.L | Supramarginal gyrus | FC+ | BPRS+ |
| Sarpal, 2016 | Seed-based | Nucleus accumbens.R | Supplemental motor area | FC- | BPRS+ |
|  |  | **Striatum** | **DMN** | **Unique connections: 16** | **total connection: 25** |
| Sarpal, 2016 | Seed-based | Nucleus accumbens.R | Middle temporalgyrus | FC- | BPRS+ |
| Sarpal, 2016 | Seed-based | Nucleus accumbens.L | Middle temporalgyrus | FC- | BPRS+ |
| Sarpal, 2016 | Seed-based | Nucleus accumbens.R | Middle temproal gyrus | FC- | BPRS+ |
| Sarpal, 2016 | Seed-based | Nucleus accumbens.R | Precuneus | Fc+ | BPRS+ |
| Sarpal, 2016 | Seed-based | Nucleus accumbens.R | Precuneus | Fc+ | BPRS+ |
| Sarpal, 2016 | Seed-based | Nucleus accumbens.R | Superior frontal gyrus, paracingulate gyrus | FC- | BPRS+ |
| Sarpal, 2016 | Seed-based | Nucleus accumbens.R | Hippocampus/parahippocampal gyrus | FC- | BPRS+ |
| Sarpal, 2016 | Seed-based | Nucleus accumbens.L | Superior parietal lobule | FC+ | BPRS+ |
| Kraguljac, 2016 | Seed-based | Caudate nucelus.R | Hippocampus (anterior).L | FC+ | BPRS+ |
| Sarpal, 2016 | Seed-based | Caudate (ventral).R | PCC | FC- | BPRS+ |
| Nelson, 2022a | Seed-based | Caudate | PCC | FC+ | BPRS+ |
| Sarpal, 2016 | Seed-based | Caudate (dorsal).R | PCC | FC+ | BPRS+ |
| Sarpal, 2016 | Seed-based | Caudate (ventral).R | Precuneus | FC- | BPRS+ |
| Sarpal, 2016 | Seed-based | Caudate (ventral).R | Precuneus | FC- | BPRS+ |
| Sarpal, 2016 | Seed-based | Caudate (dorsal).R | Angular Gyrus | FC+ | BPRS+ |
| Nelson, 2022a | Seed-based | Caudate.B | Medial prefrontal cortex | FC+ | BPRS+ |
| Nelson, 2022b | Seed-based | Caudate.B | Medial prefrontal cortex | FC+ | BPRS+ |
| Nelson, 2022a | Seed-based | Caudate.B | Temporoparietal junction.L | FC+ | BPRS+ |
| Nelson, 2022b | Seed-based | Caudate.B | Temporoparietal junction.L | FC+ | BPRS+ |
| Sarpal, 2016 | Seed-based | Putamen (dorsal caudal).R | Middle temporalgyrus | FC+ | BPRS+ |
| Sarpal, 2016 | Seed-based | Putamen (dorsal caudal).R | Middle temporalgyrus | FC+ | BPRS+ |
| Sarpal, 2016 | Seed-based | Putamen (ventral rostal).R | Precuneus | FC+ | BPRS+ |
| Nelson, 2022a | Seed-based | Putamen | Temporoparietal junction.L | FC+ | BPRS+ |
| Deng, 2022 | Seed-based | Putamen.L | Retrosplenial cortex | FC+ | SAPS |
| Hadley, 2013 | Seed-based | VTA/midbrain | Superior frontal gyrus, Middle frontal gyrus, Inferior frontal gyrus, Superior frontal gyrus, Middle frontal gyrus, ACC | FC- | BPRS total |
|  |  | **Striatum** | **VAN** | **Unique connections: 15** | **total connection: 30** |
| Han, 2019 | Seed-based | Putamen (ventral rostal).L | ACC.L | FC- | PANSS+ |
| Sarpal, 2016 | Seed-based | Putamen (ventral rostal).R | ACC | FC- | BPRS+ |
| Sarpal, 2016 | Seed-based | Putamen (ventral rostal).R | ACC | FC- | BPRS+ |
| Cadena, 2018 | Seed-based* | Putamen.L | ACC | FC+ | BPRS+ |
| Cadena, 2018 | Seed-based* | Putamen.R | ACC | FC+ | BPRS+ |
| Sarpal, 2016 | Seed-based | Putamen (ventral rostal).R | Insular cortex.R | FC- | BPRS+ |
| Sarpal, 2016 | Seed-based | Putamen (ventral rostal).R | Insular cortex.L | FC- | BPRS+ |
| Han, 2019 | Seed-based | Putamen (ventral rostal).L | Insular cortex (anterior).L | FC- | PANSS+ |
| Sarpal, 2016 | Seed-based | Putamen (dorsal caudal).L | Insula | FC- | BPRS+ |
| Sarpal, 2016 | Seed-based | Putamen (ventral rostral).L | Insula | FC- | BPRS+ |
| Han, 2019 | Seed-based | Putamen (dorsal rostal).R | Insular cortex (Anterior).R | FC+ | PANSS- |
| Sarpal, 2016 | Seed-based | Putamen (dorsal caudal).R | Insula, central opercular cortex | FC- | BPRS+ |
| Sarpal, 2016 | Seed-based | Putamen (dorsal rostal).R | Insula, central opercular cortex | FC- | BPRS+ |
| Han, 2019 | Seed-based | Putamen (dorsal caudal).R | Inferior forntal gyrus | FC+ | PANSS- |
| Han, 2019 | Seed-based | Putamen (dorsal caudal).R | Supplementary motor area | FC+ | PANSS- |
| Sarpal, 2016 | Seed-based | Putamen (dorsal rostal).L | Insula, heschl’s gyrus | FC- | BPRS+ |
| Sarpal, 2016 | Seed-based | Putamen (dorsal rostal).L | ACC, paracingulate gyrus | FC- | BPRS+ |
| Cadena, 2018 | Seed-based* | Putamen/caudate.L | ACC | FC+ | BPRS+ |
| Sarpal, 2016 | Seed-based | Caudate (ventral).R | ACC | FC- | BPRS+ |
| Nelson, 2022a | Seed-based | Caudate.B | ACC | FC+ | BPRS+ |
| Sarpal, 2016 | Seed-based | Caudate (ventral).L | Insula | FC+ | BPRS+ |
| Blazer, 2022 | Seed-based | Cadaute (dorsal).R | Insula (anterior).R | FC- | BPRS+ |
| Nelson, 2022a | Seed-based | Cadaute.B | Insula.R | FC+ | BPRS+ |
| Nelson, 2022b | Seed-based | Caduate.B | Insula.R | FC+ | BPRS+ |
| Sarpal, 2016 | Seed-based | Caudate (dorsal).R | Insula, operculum cortex | FC- | BPRS+ |
| Sarpal, 2016 | Seed-based | Caudate (dorsal).L | Planum temporale | FC+ | BPRS+ |
| Cadena, 2018 | Seed-based* | Caudate.R | ACC | FC+ | BPRS+ |
| Cadena, 2018 | Seed-based* | Caudate.L | ACC | FC+ | BPRS+ |
| Cadena, 2018 | Seed-based* | Midbrain.R | ACC | FC+ | BPRS+ |
| Hadley, 2013 | Seed-based | VTA/midbrain | ACC (dorsal) | FC+ | BPRS total |
|  |  | **Striatum** | **Thalamus** | **Unique connections 3** | **total connection: 8** |
| Sarpal, 2016 | Seed-based | Caudate (ventral).R | Thalamus | FC- | BPRS+ |
| Sarpal, 2016 | Seed-based | Caudate (ventral).R | Thalamus | FC- | BPRS+ |
| Sarpal, 2016 | Seed-based | Caudate (ventral).R | Thalamus | FC+ | BPRS+ |
| Sarpal, 2016 | Seed-based | Caudate (ventral).L | Thalamus | FC+ | BPRS+ |
| Sarpal, 2016 | Seed-based | Putamen (ventral rostral).L | Thalamus | FC+ | BPRS+ |
| Sarpal, 2016 | Seed-based | Putamen (ventral rostral).L | Thalamus | FC+ | BPRS+ |
| Sarpal, 2016 | Seed-based | Putamen (dorsal caudal).R | Thalamus | FC- | BPRS+ |
| Sarpal, 2016 | Seed-based | Nucleus Accumbens.L | Thalamus | FC+ | BPRS+ |
|  |  | **Striatum** | **FN** | **Unique connections 6** | **total connection: 8** |
| Sarpal, 2016 | Seed-based | Putamen (ventral rostal).R | Middle frontal gyrus, dorsolateral prefrontal cortex | FC+ | BPRS+ |
| Sarpal, 2016 | Seed-based | Putamen (ventral rostal).L | Prefrontal cortex (dorsolateral) | FC+ | BPRS+ |
| Sarpal, 2016 | Seed-based | Putamen (dorsal caudal).R | Prefrontal cortex (dorsolateral) | FC+ | BPRS+ |
| Sarpal, 2016 | Seed-based | Putamen (dorsal caudal).L | Prefrontal cortex (dorsolateral) | FC+ | BPRS+ |
| Sarpal, 2016 | Seed-based | Putamen (dorsal rostal).L | Middle frontal gyrus | FC+ | BPRS+ |
| Sarpal, 2016 | Seed-based | Nucleus accumbens.R | Middle frontal gyrus | FC- | BPRS+ |
| Sarpal, 2016 | Seed-based | Caudate (ventral).L | Middle frontal gyrus | FC- | BPRS+ |
| Blazer, 2022 | CCA | Caudate (dorsal).R | FN | FC- | BPRS+ |
|  |  | **Striatum** | **LN** | **Unqiue connections 6** | **total connection: 8** |
| Sarpal, 2016 | Seed-based | Putamen (ventral rostal).R | Orbitalfrontal cortex | FC- | BPRS+ |
| Sarpal, 2016 | Seed-based | Putmanen (ventral rostral).L | Orbitalfrontal cortex | FC- | BPRS+ |
| Sarpal, 2016 | Seed-based | Putmanen (dorsal rostral).R | Orbitofrontal cortex | FC- | BPRS+ |
| Sarpal, 2016 | Seed-based | Putamen (dorsal caudal).R | Frontal pole | FC- | BPRS+ |
| Sarpal, 2016 | Seed-based | Putamen (dorsal rostal).L | Orbital frontal cortex, medial frontal cortex | FC- | BPRS+ |
| Sarpal, 2016 | Seed-based | Caudate (ventral).R | Frontal pole | FC- | BPRS+ |
| Sarpal, 2016 | Seed-based | Caudate (dorsal).L | Frontal pole, orbitalfrontal cotex | FC- | BPRS+ |
| Sarpal, 2016 | Seed-based | Nucleus accumbens.R | Frontal pole | FC- | BPRS+ |
|  |  | **Striatum** | **Striatum** | **Unique connections 5** | **total connection: 5** |
| Sarpal, 2016 | Seed-based | Caudate (dorsal).L | Putamen | FC- | BPRS+ |
| Sarpal, 2016 | Seed-based | Caudate (dorsal).L | Nucleus accumbens, caudate | FC- | BPRS+ |
| Sarpal, 2016 | Seed-based | Caudate (ventral).L | Nucleus accumbens, putamen, caudate | FC- | BPRS+ |
| Sarpal, 2016 | Seed-based | Caudate (ventral).L | Nucleus accumbens, caudate | FC- | BPRS+ |
| Nelson, 2022b | Seed-based | Putamen.B | Putamen.B | FC+ | BPRS+ |
|  |  | **Striatum** | **VN** | **Unique connections: 1** | **total connection: 2** |
| Sarpal, 2016 | Seed-based | Caudate (ventral).L | Occipital cortex | FC+ | BPRS+ |
| Sarpal, 2016 | Seed-based | Caudate (dorsal).L | Occipital cortex | FC+ | BPRS+ |
|  |  | **SN** | **SN** | **Unique connections: 3** | **total connection: 3** |
| Doucet, 2018 | CCA | Superior temporalgyrus, Precentral gyrus, post-central gyrus, Heschl gyrus | Superior temporalgyrus, Pre- and post-central gyrus, Heschl gyrus | FC- | BPRS+ |
| Zhang, 2019 | ICASeed-based | Precentral gyrus.R | Precentral gyrus.R | FC- | BPRS - |
| Zhang, 2019 | ICASeed-based | Postcentral gyrus.L | Post centralgyrus.R | NA | BPRS total |
|  |  | **FN** | **FN** | **Unique connections: 1** | **total connection: 2** |
| Anticivec, 2015 | Seed-based | Prefrontal cortex | Prefrontal cortex | FC+ | PANSS total |
| Anticivec, 2015 | Seed-based | Prefrontal cortex | Prefrontal cortex | FC+ | PANSS + |
|  |  | **SN** | **DMN** | **Unique connections: 4** | **total connection: 5** |
| Doucet, 2018 | CCA | Superior temporal gyrus, Pre- and post-central gyrus, Heschl gyrus | Precuneus (Anterior), Posterior Cingulate Cortex | FC+ | BPRS total |
| Doucet, 2018 | CCA | Paracentral lobule, Pre- and post-central gyrus | Medial prefrontal cortex, Angular gyri, Inferior temporal gyrus, inferior frontal gyrus | FC- | BPRS total |
| Doucet, 2018 | CCA | Superior temporal gyrus, Pre- and post-central gyrus, Heschl gyrus | Hippocampus, Amygdala, Temporal Pole, Middle temporal gyrus, Parahippocampal gyrus, Fusiform gyrus | FC- | BPRS total |
| Doucet, 2018 | CCA | Paracentral lobule, Pre- and post-central gyrus | Precuneus (Anterior), PCC | FC- | BPRS total |
| Kraguljac, 2016 | Seed-based | auditory cortex.L | Hippocampus (posterior).L | FC+ | BPRS+ |
|  |  | **Striatum** | **NA** | **Unique connections: 17** | **total connection: 23** |
| Sarpal, 2016 | Seed-based | Putamen (ventral rostal).R | Orbital frontal cortex, subcallosal cortex, medial frontal cortex | FC- | BPRS+ |
| Sarpal, 2016 | Seed-based | Putamen (ventral rostal).R | Orbitofrontal cortex, subcallosal cortex, medial frontal cortex | FC- | BPRS+ |
| Sarpal, 2016 | Seed-based | Putamen (ventral rostal).R | Superior temporal gyrus | FC- | BPRS+ |
| Sarpal, 2016 | Seed-based | Putamen (ventral rostal).L | Subcollosal cortex | FC- | BPRS+ |
| Sarpal, 2016 | Seed-based | Putamen (dorsal rostral).R | Insula, central opercular cortex, precentral gyrus | FC- | BPRS+ |
| Sarpal, 2016 | Seed-based | Putamen (dorsal caudal).L | Insula, opercular cortex, precentral gyrus | FC- | BPRS+ |
| Sarpal, 2016 | Seed-based | Putamen (dorsal caudal).L | Insula, opercular cortex, precentral gyrus | FC- | BPRS+ |
| Sarpal, 2016 | Seed-based | Putamen (dorsal caudal).R | Insula, central opercular cortex, precentral gyrus | FC- | BPRS+ |
| Sarpal, 2016 | Seed-based | Putamen (dorsal caudal).R | Central opercular cortex | FC- | BPRS+ |
| Sarpal, 2016 | Seed-based | Putamen (dorsal rostal).L | Frontal operculum cortex, inferior frontal gyrus | FC- | BPRS+ |
| Sarpal, 2016 | Seed-based | Nucleus accumbens.R | Inferior frontal gyrus | FC- | BPRS+ |
| Sarpal, 2016 | Seed-based | Nucleus Accumbens.L | Inferior frontal gyrus | FC- | BPRS+ |
| Sarpal, 2016 | Seed-based | Nucleus accumbens.R | Superior temporal gyrus | FC- | BPRS+ |
| Sarpal, 2016 | Seed-based | Nucleus Accumbens.L | Lateral occipital cortex/Precuneus | FC+ | BPRS+ |
| Sarpal, 2016 | Seed-based | Caudate (dorsal).R | Inferior frontal gyrus | FC- | BPRS+ |
| Blazer, 2022 | Seed-based | Caudate (dorsal).R | Inferior frontal gyrus.R | FC- | BPRS+ |
| Sarpal, 2016 | Seed-based | Caudate (dorsal).L | Superior frontal gyrus | FC+ | BPRS+ |
| Sarpal, 2016 | Seed-based | Caudate (dorsal).R | Frontal opercular cortex | FC- | BPRS+ |
| Sarpal, 2016 | Seed-based | Caudate (dorsal).L | Inferior temporal gyrus | FC- | BPRS+ |
| Hadley, 2013 | Seed-based | VTA/midbrain | Middle frontal gyrus, Supplementary motor area, Paracentral lobule, Precentral gyrus, anterior cingulate cortex, Middle cingulate cortex | FC+ | BPRS total |
| Hadley, 2013 | Seed-based | VTA/midbrain | Inferior parietal gyrus, Angular gyrus, Supramarginal gyrus, Precuneus, Superior occipital gyrus, Cuneus, Calcarine fissure, Lingual gyrus, Middle cingulate cortex, Posterior cingulate cortex, Thalamus | FC- | BPRS total |
| Hadley, 2013 | Seed-based | VTA/midbrain | Middle temporal gyrus, Inferior parietal gyrus, Angular gyrus, Middle occipital gyrus | FC- | BPRS total |
| Hadley, 2013 | Seed-based | VTA/midbrain | Calcarine fissure, Fusiform/Lingual gyrus, Hippocampus/Parahippocampus, Cerebellum, Vermis | FC- | BPRS total |
|  |  | **DMN** | **VN** | **Unique connections: 2** | **total connection: 2** |
| Doucet, 2018 | CCA | Hippocampus, Amygdala, Temporal Pole, Middle temporal gyrus, Parahippocampal gyrus, Fusiform gyrus | Cuneus, Calcarine, Lingual gyri | FC+ | BPRS+ |
| Kraguljac, 2016 | Seed-based | Hippocampus (anterior).R | Lingual gyrus (Bilateral) | FC- | BPRS+ |
|  |  | **VAN** | **DMN** | **Unique connections: 3** | **total connection: 5** |
| Blessing, 2019 | ICA | Insular-opercular cortex | Hippocampus (anteromedial).L | FC+ | BPRS total |
| Blessing, 2019 | ICA | Insular-opercular cortex | Hippocampus (anteromedial).L | FC+ | BPRS positive |
| Doucet, 2018 | CCA | Insula, ACC (dorsal), Supplementary motor area, Middle frontal gyrus, Thalamus, Putamen, Pallidum | Precuneus (dorsal posterior): Precuneus (Superior parietal) | FC- | BPRS+ |
| Doucet, 2018 | CCA | Insula, ACC(dorsal), Supplementary motor area, Middle frontal gyrus, Thalamus, Putamen, Pallidum | Precuneus (dorsal Central) | FC- | BPRS+ |
| Kraguljac, 2016 | Seed-based | Anterior cingulate cortex.L | Hippocampus (Anterior).L | FC+ | BPRS+ |
|  |  | **DAN** | **DAN** | **Unique connections: 2** | **total connection: 2** |
| Kraguljac, 2015 | Seed-based | Superior parietal gyrus, Inferior parietal gyrus, cuneus/precuneus, superior and middle occipital gyrus | Superior parietal gyrus, Inferior parietal gyrus, cuneus/precuneus, superior and middle occipital gyrus | FC+ | BPRS total |
| Kraguljac, 2015 | Seed-based | Middle temporal gyrus, angular gyrus, superior parietal gyrus, cuneus/precuneus, superior and middle occipital gyrus, calcarine sulcus | Middle temporal gyrus, angular gyrus, superior parietal gyrus, cuneus/precuneus, superior and middle occipital gyrus, calcarine sulcus | FC+ | BPRS total |
|  |  | **VAN** | **SN** | **Unique connections: 1** | **total connection: 1** |
| Doucet, 2018 | CCA | Insula, ACC (dorsal), Supplementary motor area, Middle frontal gyrus, Thalamus, Putamen, Pallidum | Paracentral lobule, Pre- and post-central gyrus | FC+ | BPRS+ |
|  |  | **VN** | **VN** | **Unique connections: 1** | **total connection: 1** |
| Doucet, 2018 | CCA | Superior occipital gyrus, Middle occipital gyrus, Inferior occipital gyrus, Lingual, Fusiform gyrus | Superior occipital gyrus, Middle occipital gyrus, Inferior occipital gyrus, Lingual, Fusiform gyrus | FC- | BPRS+ |
|  |  | **NA** | **NA** | **Unique connections: 6** | **total connection: 6** |
| Doucet, 2018 | CCA | Superior occipital gyrus, Middle occipital gyrus, Inferior occipital gyrus, Lingual, Fusiform gyrus | Thalamus, Caudate, Putamen, Hippocampus | FC+ | BPRS+ |
| Doucet, 2018 | CCA | Precuneus, PCC | Thalamus, Caudate, Putamen, Hippocampus | FC+ | BPRS+ |
| Doucet, 2018 | CCA | Insula, ACC (dorsal), Supplementary motor area, Middle frontal gyrus, Thalamus, Putamen, Pallidum | Thalamus, Caudate, Putamen, Hippocampus | FC- | BPRS+ |
| Doucet, 2018 | CCA | Middle frontal gyrus, Inferior frontal gyrus, Inferior parietal gyrus | Middle frontal gyrus, Inferior frontal gyrus, Inferior parietal gyrus | FC- | BPRS+ |
| Doucet, 2018 | CCA | Thalamus, Caudate, Putamen, Hippocampus | Thalamus, Caudate, Putamen, Hippocampus | FC- | BPRS+ |
| Doucet, 2018 | CCA | Superior occipital gyrus, Middle occipital gyrus, Inferior occipital gyrus, Lingual, Fusiform gyrus | Middle frontal gyrus, Inferior frontal gyrus, Inferior parietal gyrus | FC- | BPRS+ |
|  |  | DMN | NA | **Unique connections: 5** | total connection: 6 |
| Deng, 2022 | Seed-based | PCC | Inferior frontal gyrus (orbital part).R | FC+ | SAPS |
| Deng, 2022 | Seed-based | PCC | Inferior frontal gyrus (orbital part).L | FC+ | SAPS |
| Deng, 2022 | Seed-based | Hippocampal formation | Inferior temporal gyrus.R | FC- | SAPS |
| Deng, 2022 | Seed-based | Hippocampal formation | Inferior parietal lobule.L | FC+ | SAPS |
| Deng, 2022 | Seed-based | Hippocampal formation | Inferior frontal gyrus (triangular part).L | FC+ | SAPS |
| Deng, 2022 | Seed-based | Retrosplenial Cortex | Inferior frontal gyrus (triangular part).R | FC+ | SAPS |

**Supplementary file S6 – Clusters**

**Supplementary table S7 -** Overview of clusters

**Abbreviations:** ACC: Anterior cingulate cortex

| Cluster 1 | Superior frontal gyrus, Middle frontal gyrus, Inferior frontal gyrus, Superior frontal gyrus, Middle frontal gyrus, ACC |
| --- | --- |
| Cluster 2 | Insula, central opercular cortex, precentral gyrus |
| Cluster 3 | Nucleus accumbens, putamen, caudate |
| Cluster 4 | Orbital frontal cortex, subcallosal cortex, medial frontal cortex |
| Cluster 5 | Middle frontal gyrus, Supplementary motor area, Paracentral lobule, Precentral gyrus, ACC, Middle cingulate cortex |
| Cluster 6 | Inferior parietal gyrus, Angular gyrus, Supramarginal gyrus, Precuneus, Superior occipital gyrus, Cuneus, Calcarine fissure, Lingual gyrus, Middle cingulate cortex, Posterior cingulate cortex, Thalamus |
| Cluster 7 | Middle temporal gyrus, Inferior parietal gyrus, Angular gyrus, Middle occipital gyrus |
| Cluster 8 | Calcarine fissure, Fusiform/Lingual gyrus, Hippocampus/Parahippocampus, Cerebellum, Vermis |
| Cluster 9 | Cuneus/precuneus, superior and inferior parietal lobes, lingual gyrus, middle occipital lobe, calcarine sulcus |
| Cluster 10 | Hippocampus, Amygdala, Temporal Pole, Middle temporal gyrus, Parahippocampal gyrus, Fusiform gyrus |
| Cluster 11 | Medial prefrontal cortex, Angular gyri, Inferior temporal gyrus, inferior frontal gyrus |
| Cluster 12 | Superior temporal gyrus, Precentral gyrus, post-central gyrus, Heschl gyrus |
| Cluster 13 | Cuneus, Calcarine, Lingual gyri |
| Cluster 14 | Insula, ACC (dorsal), Supplementary motor area, Middle frontal gyrus, Thalamus, Putamen, Pallidum |
| Cluster 15 | Superior occipital gyrus, Middle occipital gyrus, Inferior occipital gyrus, Lingual, Fusiform gyrus |
| Cluster 16 | Lingual gyrus, fusiform gyrus, precuneus, calcarine sulcus, cerebellum |
| Cluster 17 | Superior parietal gyrus, Inferior parietal gyrus, cuneus/precuneus, superior and middle occipital gyrus |
| Cluster 18 | Middle temporal gyrus, angular gyrus, superior parietal gyrus, cuneus/precuneus, superior and middle occipital gyrus, calcarine sulcus |

**Supplementary S7: Change FC related to AP-R**

**Supplementary table S8** - Change graph theory related to AP-R

**Abbeverations**: BPRS: Brief Psychiatric Rating Scale, DMN: Default Mode Network, LN: Limbic Network, FDR: False discovery Rate,NS: Not significant, NR: not reported, PANSS: Positive and Negative Symptom Scale, RS: Resting state.

| **First author, year of publication** | **Network characteristic** | **Location ( Network)** | **Alteration of functional connectivity associated with treatment response** | **Clinical assessment** | **R-value*** | **P-value**** |
| --- | --- | --- | --- | --- | --- | --- |
| Wang, 2019 | Clustering coefficient (global) | - | Increase | PANSS- | 0.45 | 0.04 |
| Wang, 2019 | Clustering coefficient (global) | - | Increase | PANSS+ | 0.065 | 0.960 |
| Wang, 2019 | Clustering coefficient (global) | - | Increase | PANSS general | -0.009 | 0.960 |
| Wang, 2019 | Clustering Coefficient (global) | - | Increase | PANSS total | 0.213 | 0.482 |
| Wang, 2019 | Local network efficiency | Right frontal pole (LN), superior parietal lobule (DMN) | Increase | PANSS general | 0.418 | 0.052 |
| Wang, 2019 | Local network efficiency | Right middle temporal gyrus (DMN), left cuneal cortex (DMN) | Increase | PANSS general | 0.557 | 0.005 |
| Wang, 2019 | Local network efficiency | Right middle temporal gyrus DMN), left cuneal cortex (DMN) | Increase | PANSS total | 0.540 | 0.005 |
| Hadley, 2016 | Clustering coefficient (global) | - | Decrease | BPRS total | NR | NR |
| Hadley, 2016 | Clustering coefficient (global) |  | NS | BPRS+ | NR, NS | NR, NS |
| Hadley, 2016 | Clustering coefficient (global) |  | NS | BPRS - | NR, NS | NR, NS |
| Hadley, 2016 | Efficiency (global) | - | NS | BPRS total | NR,NS | NR, NS |
| Hadley, 2016 | Efficiency (global) |  | Increase | BPRS total | NR | NR |
| Liu,2022 | Degree centrality | Left putamen (Striatum) | Decrease | PANSS total | -0.51 | P<0.001 |
| Liu,2022 | Degree centrality | Right putamen(Striatum) | Decrease | PANSS total | -0.52 | P<0.001 |
| Liu,2022 | Degree centrality | Bilateral putamen(Striatum) | Decrease | PANSS+ | -0.49 | P<0.001 |

*r was calculated using partial correlation

**p was corrected for multiple correlations using FDR

**Supplementary table S9 –** Change FC related to AP-R

**Abbeverations:** ICA: Independent component analysis, FC: Functional connectivity, FC+: positive correlations between signals FC-: anticorrelated signals, BPRS: Brief Psychiatric Rating Scale, PANSS: Positive and Negative Syndrome Scale, RSN: Resting State Network, DMN: Default mode network, VAN: Ventral attention network, SN: Sensorymotor network. Structures: ACC: Anterior cingulate cortex, PCC: Posterior Cingulate cortex

| First author, year of publication | Analytical approach | Region A | Region B | Direction of FC-change between A and B | Clinical assesment |
| --- | --- | --- | --- | --- | --- |
|  |  | **Striatum** | **VAN** | **Unique connections 5** | **Total connections: 6** |
| Cadena, 2018 | Seed-based* | Putamen (l) | ACC (dorsal) (b) | Increase | PANSS+ |
| Sarpal, 2015 | Seed-based | Putamen (ventral rostral) (r) | ACC (b) | Increase | BPRS+ |
| Sarpal, 2015 | Seed-based | Putamen (ventral rostral) (r) | Insula.R | Increase | BPRS+ |
| Han, 2020 | Seed-based | Putamen (dorsal rostral) (r) | Anterior insular cortex (b) | Decrease | PANSS- |
| Sarpal, 2015 | Seed-based | Caudate (dorsal) (r) | ACC (b) | Increase | BPRS+ |
| Blazer, 2022 | Seed-based | Caudate (dorsal).R | Anterior insular cortex.R | Increase | BPRS+ |
|  |  | **Striatum** | **DAN** | **Unique connections 3** | **Total connections: 4** |
| Sarpal, 2015 | Seed-based | Caudate (ventral)/nucleus accumbens (r) | Superior parietal lobule (l) | Decrease | BPRS+ |
| Sarpal, 2015 | Seed-based | Caudate (ventral)/nucleus accumbens (r) | Superior parietal lobe (r) | Decrease | BPRS+ |
| Sarpal, 2015 | Seed-based | Caudate (ventral)/nucleus accumbens (r) | Supramarginal gyrus (r) | Decrease | BPRS+ |
| Sarpal, 2015 | Seed-based | Caudate (ventral) (l) | Superior parietal lobe (r) | Decrease | BPRS+ |
|  |  | **Striatum** | **SN** | **Unique connections 2** | **Total connections: 2** |
| Sarpal, 2015 | Seed-based | Caudate (ventral)/nucleus accumbens (r) | Supramarginal gyrus (l) | Decrease | BPRS+ |
| Han, 2020 | Seed-based | Putamen (dorsal caudal) (r) | Supplementary motor area (r) | Decrease | PANSS- |
|  |  | **Striatum** | **Thalamus** | **Unique connections 2** | **Total connections: 2** |
| Sarpal, 2015 | Seed-based | Caudate (dorsal) (r) | Thalamus (l) | Increase | BPRS+ |
| Hadley, 2014 | Seed-based | VTA/midbrain | Thalamus | NS | BPRS general |
|  |  | **Striatum** | **LN** | **Unique connections 1** | **Total connections: 1** |
| Sarpal, 2015 | Seed-based | Caudate (dorsal) (r) | Orbitofrontal cortex (r) | Increase | BPRS+ |
|  |  | **Striatum** | **FN** | **Unique connections 2** | **Total connections: 2** |
| Sarpal, 2015 | Seed-based | Caudate (dorsal) (r) | Dorsolateral prefrontal cortex (r) | Increase | BPRS+ |
| Blazer, 2022 | CCA | Caudate (dorsal).R | FN | Increase | BPRS+ |
|  |  | **FN** | **FN** | **Unique connections 1** | **Total connections: 3** |
| Anticivec, 2015 | Seed-based | Prefrontal cortex | Prefrontal cortex | Decrease | PANSS total |
| Anticivec, 2015 | Seed-based | Prefrontal cortex | Prefrontal cortex | Decrease | PANSS + |
| Anticevic, 2015 | Seed-based | Prefrontal cortex | Prefrontal cortex | Decrease | PANSS negative |
|  |  | **Striatum** | **DMN** | **Unique connections 4** | **Total connections: 5** |
| Sarpal, 2015 | Seed-based | Caudate (ventral)/nucleus accumbens (r) | Hippocampus (l) | Increase | BPRS+ |
| Kraguljac, 2016 | Seed-based | Caudate | Hippocampus (posterior) (r) | Increase | BPRS+ |
| Kraguljac, 2016 | Seed-based | Caudate (r) | Hippocampus (anterior) (r) | Decrease | BPRS+ |
| Han, 2020 | Seed-based | Putamen (ventral rostal) (l) | Superior frontal gyrus (l) | Increase | PANSS+ |
| Deng, 2022 | Seed-based | Putamen.L | Retrosplenial cortex | Decrease | SAPS |
|  |  | **Striatum** | **NA** | **Unique connections 1** | **Total connections: 1** |
| Blazer, 2022 | Seed-based | Caudate (dorsal).R | Inferior frontale lobe.R | Increase | BPRS+ |
|  |  | **DMN** | **DMN** | **Unique connections 6** | **Total connections: 6** |
| Duan, 2020 | Seed-based | PCC | Middle temporal gyrus | NS | PANSS total |
| Duan, 2020 | Seed-based | PCC | Parahippocampal gyrus | NS | PANSS total |
| Li, 2016 | Seed-based | Inferior parietal lobe (r) | Inferior parietal lobe.L | NS | PANSS general |
| Zong, 2018 | ICA | PCC/precuneus | medial prefrontal cortex | Increase | PANSS+ |
| Deng, 2022 | Seed-based | PCC | Precuneus.L | Increase | SAPS |
| Shan, 2021 | Seed-based | Superior/middle medial prefrontal cortex | Superior/middle medial prefrontal cortex | NS | PANSS general |
|  |  | **DMN** | **VN** | **Unique connections 3** | **Total connections: 5** |
| Wang, 2018 | Seed-based | Middle temporal gyrus (r) | Cuneal gyrus (l) | Increase | PANSS general |
| Wang, 2018 | Seed-based | Middle temporal gyrus (r) | Cuneal gyrus (l) | Increase | PANSS total |
| Kraguljac, 2016 | Seed-based | Hippocampus (posterior) (r) | Lingual gyrus | Increase | BPRS+ |
| Kraguljac, 2016 | Seed-based | Hippocampus (posterior) (r) | Lingual gyrus | Increase | BPRS+ |
| Duan, 2020 | Seed-based | PCC | Lingual gyrus | Increase | PANSS total |
|  |  | **DMN** | **SN** | **Unqiue connections 3** | **Total connections: 5** |
| Kraguljac, 2016 | Seed-based | Hippocampus (anterior) (r) | Auditory cortex (l) | Decrease | BPRS+ |
| Kraguljac, 2016 | Seed-based | Hippocampus (anterior) (l) | Auditory cortex (l) | Decrease | BPRS+ |
| Kraguljac, 2016 | Seed-based | Hippocampus (posterior) (l) | Auditory cortex (l) | Decrease | BPRS+ |
| Duan, 2020 | Seed-based | PCC | Superior temporal gyrus | NS | PANSS total |
| Duan, 2020 | Seed-based | PCC | Precentral gyrus | NS | PANSS total |
|  |  | **DMN** | **Thalamus** | **Unique connections 1** | **Total connections: 1** |
| Duan, 2020 | Seed-based | PCC | Thalamus | NS | PANSS total |
|  |  | **DMN** | **NA** | **Unique connections: 8** | **Total connections: 9** |
| Duan, 2020 | Seed-based | PCC | Cerebellum posterior lobe | NS | PANSS total |
| Duan, 2020 | Seed-based | PCC | Medial frontal gyrus | NS | PANSS total |
| Kraguljac, 2016 | Seed-based | Hippocampus (posterior) (r) | Medial prefrontal cortex/ACC | Increase | BPRS+ |
| Deng, 2022 | Seed-based | PCC | Inferior frontal gyrus (orbital part).R | Decrease | SAPS |
| Deng, 2022 | Seed-based | PCC | Inferior frontal gyrus (orbital part).L | Decrease | SAPS |
| Deng, 2022 | Seed-based | Retrosplenial Cortex | Inferior frontal gyrus (triangular part).R | Decrease | SAPS |
| Deng, 2022 | Seed-based | Hippocampal formation | Inferior temporal gyrus.R | Decrease | SAPS |
| Deng, 2022 | Seed-based | Hippocampal formation | Inferior parietal lobule.L | Decrease | SAPS |
| Deng, 2022 | Seed-based | Hippocampal formation | Inferior frontal gyrus (triangular part).L | Decrease | SAPS |
|  |  | **DMN** | **VAN** | **Unique connections 2** | **Total connections: 4** |
| Blessing, 2019 | ICA | Hippocampus (anteromedial) (l) | Insular opercular cortex (posterior) (r) | Decrease | BPRS total |
| Blessing, 2019 | ICA | Hippocampus (anteromedial) | Insular opercular cortex | Decrease | BPRS positive |
| Li, 2020 | Seed-based | ACC (r) | ACC (l) | NS | PANSS general |
| Li, 2020 | Seed-based | ACC (r) | ACC (l) | NS | PANSS+ |
|  |  | **DAN** | **DAN** | **Unique connections: 1** | **Total connections: 1** |
| Kraguljac, 2015 | Seed-based | Lingual gyrus, fusiform gyrus, precuneus, clacarine sulcus, cerebellum | Lingual gyrus, fusiform gyrus, precuneus, clacarine sulcus, cerebellum | Decrease | BPRS total |

*Task-based: Stroop color naming task
